# Widespread Regulation of miRNA Biogenesis at the Dicer Step by the Cold-Inducible RNA-Binding Protein, RBM3

**DOI:** 10.1371/journal.pone.0028446

**Published:** 2011-12-01

**Authors:** Julie Pilotte, Esther E. Dupont-Versteegden, Peter W. Vanderklish

**Affiliations:** 1 Department of Neurobiology, The Scripps Research Institute, La Jolla, California, United States of America; 2 Department of Rehabilitation Sciences, College of Health Sciences, University of Kentucky, Lexington, Kentucky, United States of America; ETH Zurich, Switzerland

## Abstract

MicroRNAs (miRNAs) play critical roles in diverse cellular events through their effects on translation. Emerging data suggest that modulation of miRNA biogenesis at post-transcriptional steps by RNA-binding proteins is a key point of regulatory control over the expression of some miRNAs and the cellular processes they influence. However, the extent and conditions under which the miRNA pathway is amenable to regulation at posttranscriptional steps are poorly understood. Here we show that RBM3, a cold-inducible, developmentally regulated RNA-binding protein and putative protooncogene, is an essential regulator of miRNA biogenesis. Utilizing miRNA array, Northern blot, and PCR methods, we observed that over 60% of miRNAs detectable in a neuronal cell line were significantly downregulated by knockdown of RBM3. Conversely, for select miRNAs assayed by Northern blot, induction of RBM3 by overexpression or mild hypothermia increased their levels. Changes in miRNA expression were accompanied by changes in the levels of their ∼70 nt precursors, whereas primary transcript levels were unaffected. Mechanistic studies revealed that knockdown of RBM3 does not reduce Dicer activity or impede transport of pre-miRNAs into the cytoplasm. Rather, we find that RBM3 binds directly to ∼70 nt pre-miRNA intermediates and promotes / de-represses their ability as larger ribonucleoproteins (pre-miRNPs) to associate with active Dicer complexes. Our findings suggest that the processing of a majority of pre-miRNPs by Dicer is subject to an intrinsic inhibitory influence that is overcome by RBM3 expression. RBM3 may thus orchestrate changes in miRNA expression during hypothermia and other cellular stresses, and in the euthermic contexts of early development, differentiation, and oncogenesis where RBM3 expression is highly elevated. Additionally, our data suggest that temperature-dependent changes in miRNA expression mediated by RBM3 may contribute to the therapeutic effects of hypothermia, and are an important variable to consider in *in vitro* studies of translation-dependent cellular events.

## Introduction

MicroRNAs (miRNAs) are a family of short, noncoding RNAs that regulate translation of mRNAs by mechanisms involving the binding of complementary sequences [1], [2]. The influence of miRNAs on the proteome and cellular events is extensive as they regulate an estimated 60% of the transcriptome [3] and play key roles in differentiation, plasticity, circadian rhythm, immunity, and disease [4]–[10]. The post-transcriptional biogenesis of most miRNAs involves a sequential cleavage process mediated by RNase III family enzymes (reviewed in ref. [11]). Primary transcripts (pri-miRNAs) are first cleaved by Drosha in the nucleus to yield ∼70 nt hairpin precursors (pre-miRNAs). These intermediates are transported to the cytoplasm where ∼22mer dsRNAs are excised by Dicer. Typically, one strand of the dsRNAs is inserted as a mature miRNA into the RNA-induced silencing complex (RISC), which contains members of the Argonaute (Ago) protein family that contribute to translational regulation [12], [13]. Recent studies indicate that some RNA-binding proteins (RNA-BPs) can regulate discrete processing steps [3], differentially blocking [14], [15] or promoting [16]–[18] the formation of specific miRNAs to control cellular proliferation and differentiation. Elegant examples of this mechanism include the attenuation of let-7 biogenesis in embryonic stem cells by the pluripotency factor LIN28 [15], [19], and the selective enhancement of miR-18a biogenesis from a polycistronic transcript by hnRNPA1 [16].

RBM3 is a member of a small, highly conserved family of RNA-BPs that is upregulated in response to mild hypothermia [20]. Members of this family have been proposed to play an adaptive role by acting as mRNA chaperones that preserve translation capacity, or enhance translation rates upon restoration of euthermic conditions [21]–[23]. RBM3 is ubiquitously expressed and it is the only transcript upregulated in all tissues during torpor [21]. Upregulation of RBM3 also occurs in response to other cellular stressors, such as hypoxia and degenerative conditions, where it may attenuate both apoptosis and necrosis [24], [25]. Increased expression of RBM3 has been noted in several cancer cell types where it has been proposed to act as a protooncogene that facilitates cell division and attenuates apoptosis [26]. Under normal physiological conditions, RBM3 is developmentally regulated in brain, and in the adult brain it is highly expressed in progenitor cell fields and other regions with high cerebral translation rates [27]. Taken together, these observations suggest that RBM3 may have a fundamental function in all cells that becomes of adaptive value under conditions of cellular stress, and of pathological significance in cell transformation. In prior work, we demonstrated that overexpression of RBM3 in neuronal cell lines reduces the levels of a miRNA-containing ribonucleoprotein (miRNP) peak resolved on sucrose gradients [22], [23]. This observation suggested that the functions of RBM3 - in stress adaptation, normal physiology, and potentially in oncogenesis – may involve the miRNA pathway.

In the present study, we demonstrate that RBM3 regulates the posttranscriptional biogenesis of a majority of miRNAs at the Dicer step. We present evidence that it does this by binding ∼70 nt miRNA precursors and promoting their ability to engage catalytically active Dicer complexes. The significance of our findings is discussed in terms of how pre-miRNAs are regulated as larger ribonucleoprotein complexes, and in terms of the set of physiological conditions that may influence the miRNA pathway through induction or suppression of RBM3 expression.

## Results

### Microarray profiling reveals broad and differential effects of RBM3 on miRNA expression

**Figure 1:**
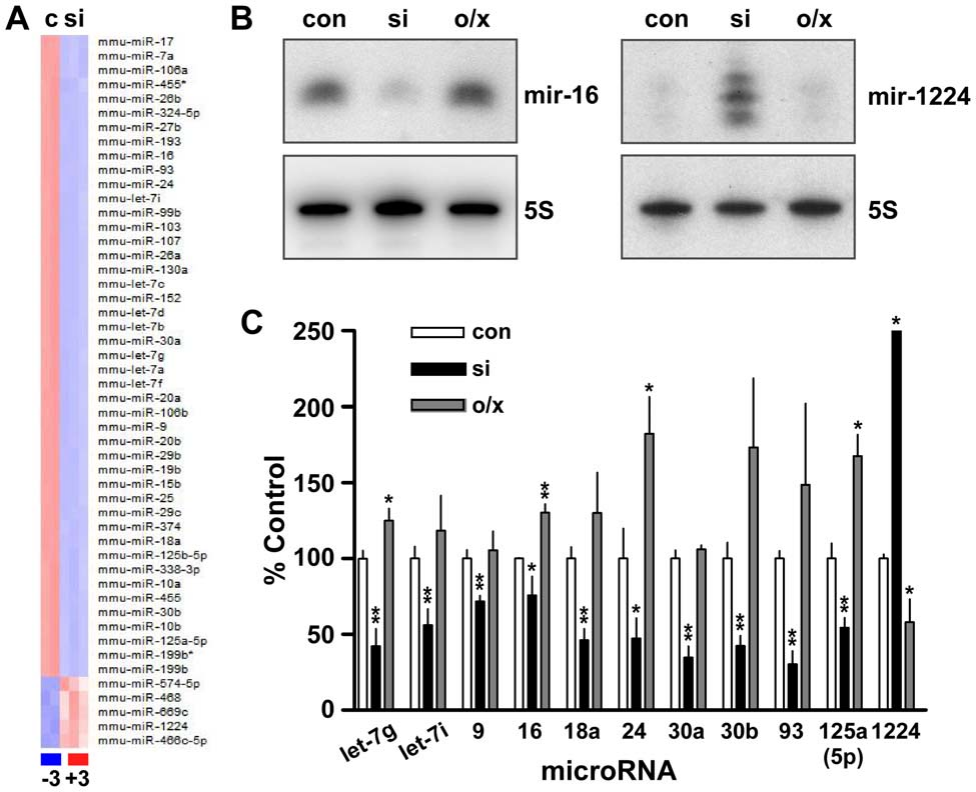
RBM3 modulates miRNA expression. (**A**) Heat map representing the top 50 significant miRNA expression differences between control (con) and RBM3 knockdown (si) B104 cells. (**B**) Northern blots of select miRNAs under con, si, and RBM3 overexpression (o/x) conditions; 5S RNA, loading control. (**C**) Group Northern blot data for RBM3-associated changes in 11 miRNAs, each normalized to 5S RNA (n = 3–5 independent experiments; * p<0.05, ** p<0.01, 1-tailed t-test).

**Figure 2:**
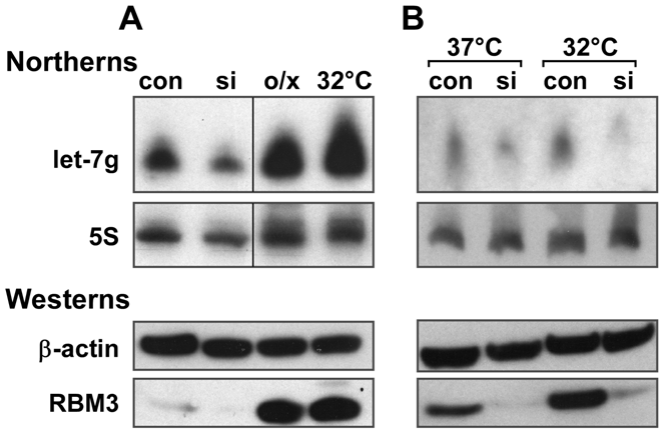
Induction of RBM3 by mild hypothermia mimics the effects of RBM3 overexpression on let-7g levels. (**A**) Upper panels: Northern blots of let-7g in control (con), RBM3 knockdown (si), RBM3 overexpression (o/x), and mildly hypothermic (32°C×24 hrs) conditions. 5S RNA is the loading control. Lower two panels: Western blots of RBM3 in the four conditions; β-actin is the loading control. (**B**) Upper panels: Northern blot of let-7g in control and RBM3 knockdown B104 cells maintained at normal (37°C) and mildly hypothermic (32°C) conditions; 5S RNA is the loading control. Lower panels: Western blot showing induction of RBM3 at 32°C in controls, but greatly attenuated expression at both temperatures in the siRNA condition. β-actin is the loading control. Enhanced expression of let-7g at 32°C is prevented when RBM3 is knocked down.

To characterize the influence of RBM3 on the miRNA pathway, we began by analyzing miRNA expression profiles after siRNA-mediated knockdown of RBM3 in the B104 neuronal cell line. Using Agilent miRNA arrays encompassing all known mouse miRNAs, we observed that a >90% reduction of RBM3 (**Figure S1**) resulted in extensive and differential changes in miRNA expression: 129 were reduced, while 30 were increased (**Figure 1A**, **Table S1**). The set of 159 miRNAs exhibiting significant expression changes comprised 74% of the total number of miRNAs detected (216) above background threshold in B104 cells. Within this set were miRNAs previously implicated in proliferation and migration (e.g. miR-9 [28]), differentiation (e.g. the let-7 family [29]), synaptic plasticity (e.g. miR-134 [30]), apoptosis (e.g. miR-29b [31]), oncogenesis (e.g. the miR-17-92 cluster [7], [8]) and other functions. Expression changes identified by microarray were reliably confirmed in Northern blot analyses in which we also evaluated the effects of RBM3 overexpression. Representative Northern blots for miR-16, let-7g, and miR-1224 are shown in **Figures 1B** and **2A** (with additional examples in **Figures S2, S3**). These assays revealed that knockdown and overexpression of RBM3 had opposite effects on miRNA expression. Levels of miR-16 and let-7g, for example, varied positively with the level of RBM3, while miR-1224 showed the opposite pattern. Group Northern blot data summarizing the differential and bidirectional regulation of 11 miRNAs by RBM3 is shown in **Figure 1C**.

miRNAs showing significant down regulation by microarray analysis were consistently validated by Northern blot. However, changes in miRNAs that by microarray analysis were elevated after RBM3 knockdown – 30 in total – were harder to validate. In the case of miR-1224, which is predicted to be a mirtron [32], [33] and thus processed along a pathway that does not require Drosha, large increases were validated by Northern blot. Others in the set of 30 miRNAs elevated in array analyses after RBM3 knockdown were either more variable or harder to detect in Northern blot assays. Those with very low expression may be subject to false positive variation, which would indicate that an even greater majority of the miRNA pool varies directly with RBM3 expression levels.

### Physiological induction of RBM3 by mild hypothermia enhances let-7 biogenesis in a RBM3-dependent manner

Experimental overexpression of RBM3 increased the levels of many miRNAs, and physiological induction of RBM3 by mild hypothermia [20] recapitulated this effect. Incubation of B104 cells at 32°C for 24 hours resulted in upregulation of mature let-7g (**Figure 2A**) and other miRNAs (**Figure S3**). The enhancement of let-7g steady state levels at 32°C was blocked by knockdown of RBM3, suggesting that induction of the RNA-BP was absolutely required for hypothermia related upregulation of let-7g (**Figure 2B**). These observations suggest that adaptation to cold stress may involve RBM3-mediated regulation of the miRNA pathway.

### Knockdown of RBM3 selectively impairs the processing of pre-miRNAs at the Dicer step

**Figure 3:**
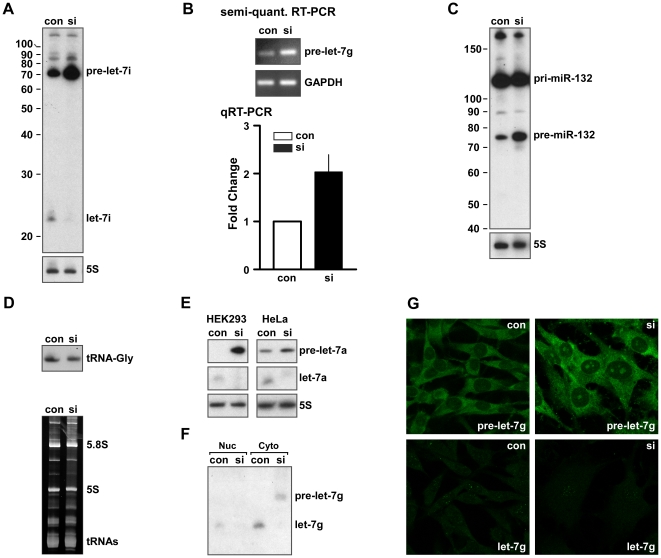
Pre-miRNA processing is selectively impaired by knockdown of RBM3. (**A**) Northern blot of let-7i showing relative levels of mature and pre-miRNA species in B104 cells under control (con) and RBM3 knockdown (si) conditions; 5S RNA, loading control. (**B**) Upper panel: Ethidium bromide stained gel showing pre-let-7g products from semi-quantitative RT-PCRs using control and RBM3 knockdown B104 cell RNA templates; GAPDH was used as a control. Lower panel: Graph illustrating fold increase in pre-let-7g after RBM3 knockdown as measured by quantitative RT-PCR (qRT-PCR; n = 3, p<0.01); U6 was used as the internal reference. (**C**) Northern blot of miR-132 showing the relative levels of pri- and pre-miR-132 species in control and RBM3 knockdown conditions. 5S RNA, loading control. (**D**) Upper panel: Northern blot of the same samples using a probe for tRNA-Gly. Lower panel: Ethidium bromide (EtBr) visualization of tRNAs. (**E**) Northern blots of let-7a in HEK 293T and HeLa cells showing attenuated processing of pre-let-7a after RBM3 knockdown. (**F**) Northern blot of let-7g in nuclear and cytoplasmic fractions from B104 cells; pre-let-7g accumulates in the cytoplasm after knockdown of RBM3. (**G**) *In situ* hybridization with probes selective for pre-let-7g (top panels) and mature let-7g (lower panels) in B104 cells under con and si conditions.

We next investigated the mechanisms by which RBM3 regulates miRNA biogenesis. RBM3 has a relatively simple domain structure, being comprised of a single RNA-recognition motif (RRM) and a c-terminal region rich in arginine, glycine, and tyrosine. Because many RNA-BPs harboring these domains (particularly hnRNPs) regulate multiple aspects of RNA metabolism, we considered that RBM3 may act at several steps in the process of miRNA biogenesis, including transcription, splicing, transport, and stability. In addition, recent studies on the roles of the RNA-BPs hnRNPA1, LIN28, and KSRP in the biogenesis of specific sets of miRNAs have demonstrated that post-transcriptional cleavage of pre-miRNAs can also be regulated [15]–[17]. To begin evaluating these possibilities, we used RNA probes to measure levels of mature miRNAs and their precursor species by Northern blot. Northern blots of mature let-7i and its pre-miRNA revealed that, concomitant with reduction of mature 22mers, there was an accumulation of ∼70 nt pre-miRNAs after RBM3 knockdown (**Figure 3A**). This pattern was evident for all other miRNAs examined by Northern blot (e.g. **Figures S1, S2, S3**). Elevated levels of pre-miRNAs after RBM3 knockdown were also seen by Northern blot when probes complimentary to the full length sequence of the precursor were used (**Figure S2**). To confirm this result using another method, we performed semi-quantitative RT-PCR (**Figure 3B**) and quantitative RT-PCR (qRT-PCR), both of which showed large elevations in pre-miRNAs after RBM3 knockdown. The accumulation of pre-miRNAs after RBM3 knockdown was reproducible in other cell lines. In HEK 293T and HeLa cells, the processing of pre-let-7a to mature let-7a was greatly attenuated under conditions of reduced RBM3 expression (**Figure 3E**) and accompanied by large increases in this precursor.

In contrast to pre-miRNAs, the levels of primary transcripts did not appear to change after RBM3 knockdown, suggesting that accumulation of ∼70 nt pre-miRNAs was not simply due to an increase in transcription of pri-miRNA Drosha substrates. This pattern, reminiscent of pre-miRNA accumulation when Dicer is inactive or absent [34], is seen on Northern blots of miR-132 for which both the pri- and pre-miRNA species are readily resolved on a single gel (**Figure 3C**). The large and selective increases in pre-miRNAs that we observed by Northern blot (and RT-PCR) after RBM3 knockdown did not involve changes in tRNAs that are of approximately the same size as pre-miRNAs and have hairpin structures (**Figure 3D** and **Figure S3**). Neither did it appear that the effects of RBM3 on pre-miRNAs could be due to altered splicing. While a Drosha-independent mechanism has been identified for the formation of pre-miRNAs that involves splicing of some intron-positioned miRNAs (mirtrons [32], [33]), this applies to a relative few primary transcripts and the genomic locations of the vast majority of RBM3-regulated miRNAs are not suitable for this pathway.

It is noteworthy that in our Northern blot studies of miRNA expression in B104 cells the relative levels of pre-miRNAs compared to mature ∼22mers was higher than often seen. Given that changes in pre-miRNA levels following RBM3 knockdown were confirmed by three different approaches, and that tRNAs are not affected by manipulation of RBM3, it is likely that ratios of pre-miRNAs to mature ∼22mers vary with cell type. Consistent with this idea, our own studies using HeLa and HEK293 cells (**Figure 3E**) reveal different ratios of pre-miRNAs to mature ∼22mers at steady state compared the B104 neuronal cell line and to each other. This may reflect different basal rates of pre-miRNA processing in different cell lines. Even with such differences in steady state processing, knockdown of RBM3 had the same effect in all cell lines tested: it caused selective accumulation of pre-miRNAs. This effect appeared greatest in HEK293 cells, where the steady state ratio of pre-miRNA to mature ∼22mer was the lowest of the three cell lines tested.

### Knockdown of RBM3 does not impair transport of pre-miRNAs into the cytoplasm

One plausible basis for the correlated changes in miRNAs and their parent pre-miRNAs is that RBM3 knockdown leads to a deficit in the transport of pre-miRNAs to the cytoplasm where they become a substrate for Dicer. However, while our prior work [23], [27] demonstrated that RBM3 can traffic between nuclear and cytoplasmic compartments, analysis of the subcellular distribution of pre-let-7g by Northern blot (**Figure 3F**) and *in situ* hybridization (**Figure 3G**) did not reveal deficits in cytoplasmic transport of the precursor when RBM3 was depleted. Rather, pre-miRNAs accumulated in the cytoplasm after RBM3 knockdown. There was no obvious change in mature let-7 localization after RBM3 knockdown (**Figure 3G**). Some nucleolar hybridization was seen, consistent with reports that several miRNAs are present in this compartment [35].

### Knockdown of RBM3 does not lead to reduced Dicer activity or to modifications in pre-miRNAs that impair their ability to act as Dicer substrates

**Figure 4:**
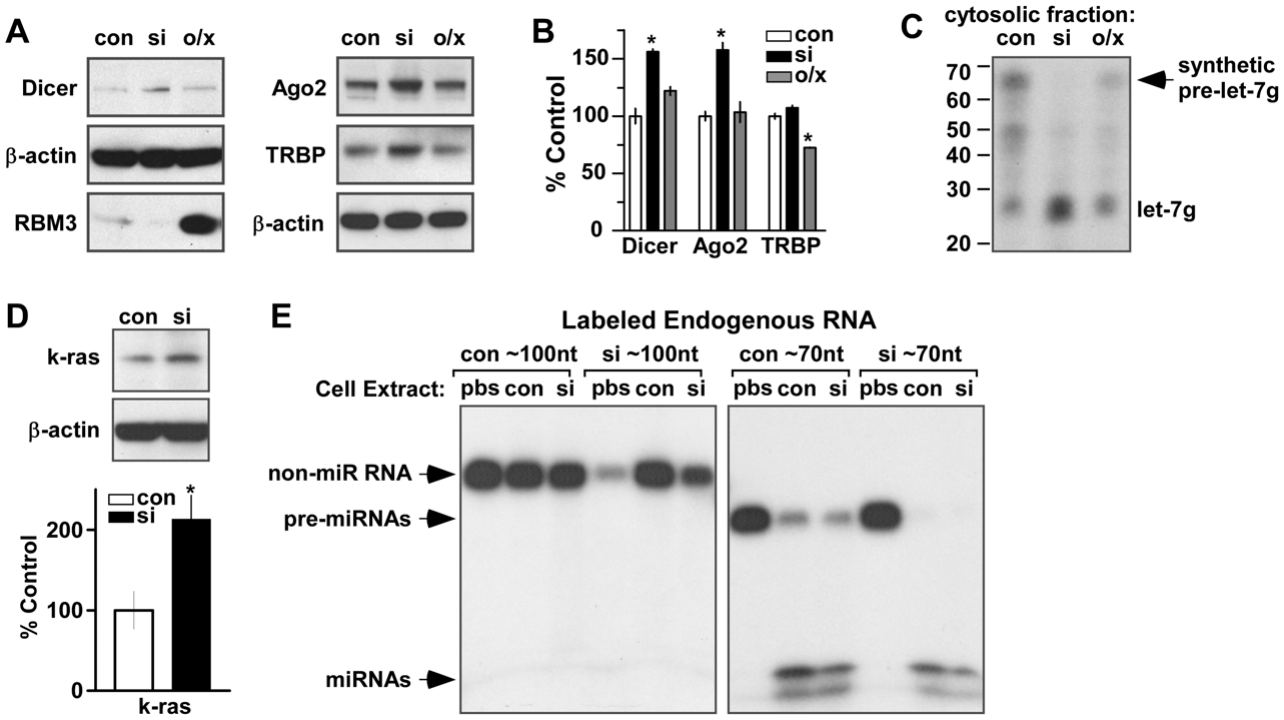
Knockdown of RBM3 does not reduce Dicer complex components or the ability of pre-miRNAs to act as substrates. (**A**) Western blots of Dicer, Ago2 and TRBP in B104 cells under control (con) conditions, and after knockdown (si) and overexpression (o/x) of RBM3. β-actin is the loading control. (**B**) Bar graph summarizing changes in Dicer (n = 4), Ago2 (n = 5), and TRBP (n = 3); * p<0.01, 2-tailed paired t-test. (**C**) Northern blots showing the processing of a synthetic pre-let-7g by cytosolic lysates from con, si, and o/x conditions. (**D**) Bar graph and example Western blot showing levels of the let-7 target, k-ras, in con and si conditions (* p<0.05, 1-tailed paired t-test, n = 3). β-actin is the loading control. (**E**) Autoradiographs showing the processing of endogenous small RNAs of ∼100 nt and ∼70 nt that were purified from B104 cells under control (con) and si conditions, end labeled, and then re-incubated with cell extracts from the indicated treatment conditions.

In light of the aforementioned findings, a logical hypothesis for the effects of RBM3 on the expression of miRNAs is that it modulates the posttranscriptional processing of pre-miRNAs by Dicer. In principle, this could involve changes in the Dicer complex, modification of pre-miRNAs, or the interaction of these entities. As RBM3 is known to promote the stability of some mRNAs [26] and overall translation [22], [23], a straightforward hypothesis is that levels of Dicer are reduced by RBM3 depletion. Unexpectedly, however, expression of Dicer was significantly elevated after RBM3 knockdown (**Figure 4A, B**). In accord with this observation, Dicer-mediated processing of a synthetic version of pre-let-7g was actually greater in lysates from RBM3 knockdown cells than in control lysates (**Figure 4C**). We next looked for deficits in two cofactors of Dicer involved in pre-miRNA processing and insertion of mature miRNAs into the RISC complex: the transactivator responsive RNA binding protein 2 (TRBP) and Ago2 [13], [36]. However, Ago2 was also expressed at significantly higher levels when RBM3 was depleted, while TRBP exhibited little change (**Figure 4A, B**). These effects were also seen in HeLa cells (**Figure S4**). These results did not support the idea that reduction of RBM3 levels leads to an insufficiency of Dicer complexes that would impair pre-miRNA processing. However, they may be consistent with downstream effects of RBM3-related changes in miRNA expression. A number of miRNAs predicted to target messages encoding Dicer complex components are reduced after RBM3 knockdown, and it has been reported that Dicer is subject to feedback regulation by let-7 [37]. We observed that all let-7 family members were reduced after RBM3 knockdown (**Table S1;** **Figure 2**, **Figures S1, S2, S3**). This suggested that known targets of let-7, including Dicer, would be upregulated even though reductions of RBM3 lead to decreased overall translation [23]. Consistent with this idea, we observed an increase in the expression of another let-7 target, k-ras [38], by Western blot after knockdown of RBM3 (**Figure 4D**).

In contrast to the effects of RBM3 knockdown in B104 cells, overexpression of the RNA-BP had no effect on Dicer or Ago2 levels and significantly decreased the expression of TRBP (**Figure 4A, B**). These results suggested that the enhanced biogenesis of many miRNAs seen when RBM3 is overexpressed does not rely on increases in Dicer complex levels or activity. Dicer and Ago2 were modestly decreased by this manipulation in HeLa cells (**Figure S4**).

### Knockdown of RBM3 does not alter the ability of pre-miRNAs to act as Dicer substrates

Altered pre-miRNA processing in the context of low RBM3 levels could also stem from modifications of precursors that alter their ability to act as Dicer substrates. Precedent for such a mechanism comes from the inhibitory effect of LIN28 on the processing of let-7 family members, which involves polyuridylation of precursors [39], [40]. As a general test of this possibility, we purified endogenous small RNAs (∼60 nt to ∼100 nt) from control and RBM3 knockdown cells and end-labeled them so that their processing could be visualized after re-incubation with cytosolic extracts from each condition (**Figure 4E**). Purified ∼70 nt RNAs from control and siRNA-treated cells were efficiently processed by both cellular extracts into mature miRNAs. There appeared to be an enhanced processing of ∼70 nt RNAs isolated from RBM3 knockdown cells by each extract (relative to control ∼70 nt RNAs); this may be due to a greater proportion of pre-miRNAs in the ∼70 nt pool of RNAs isolated from RBM3 knockdown cells. As a control for general RNase activity, end-labeled RNAs of ∼100 nt were not processed by either extract. Combined with the lack of an obvious size change in electrophoretically resolved pre-miRNAs (**Figure 3A–C**, **Figures S1, S2, S3**), these observations suggest that low RBM3 expression does not result in primary structural modifications of pre-miRNAs that impair their cleavage by Dicer.

### Evidence for a role of RBM3 in directly regulating pre-miRNA ribonucleoprotein complexes

**Figure 5:**
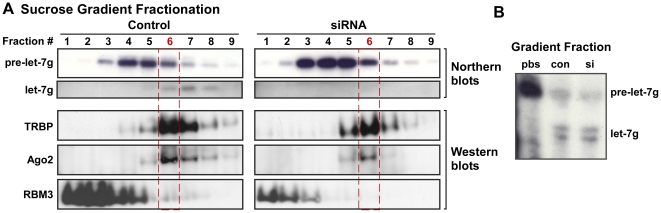
RBM3 regulates pre-miRNP composition. (**A**) Northern and Western blots showing the distribution of pre-let-7g, mature let-7g, TRBP, Ago2, and RBM3 in early (RNP) fractions from sucrose gradients of control and RBM3 siRNA-treated B104 cells. Fraction 6 (red) represents the peak of TRBP and Ago2 distribution in both conditions; pre-let-7g accumulates as a lower molecular weight pre-miRNP complex in the siRNA condition. Mature let-7g is present in TRBP / Ago2-containing fractions from control cells, but barely detectable in the same fractions from RBM3 knockdown cells. (**B**) Autoradiogram showing processing of labeled synthetic pre-let-7g in concentrated samples from the TRBP/Ago2-containing fractions of control and RBM3 knockdown conditions. Processing activity towards an exogenous precursor is still intact in the knockdown condition, even as processing of the endogenous pre-let-7g is greatly attenuated.

While lowering RBM3 expression impaired the processing of many endogenous pre-miRNAs, exogenously added precursors (synthetic or purified endogenous) were nevertheless processed efficiently in this condition. We hypothesized that endogenous and exogenous precursors differ in their ability to access Dicer, perhaps because exogenous precursors do not acquire key factors that normally limit such interactions. As expected from our *in vitro* processing data and Dicer expression results, the association of Dicer, TRBP, and Ago2 with biotinylated pre-miRNA probes (let-7i and miR-16) was actually enhanced after knockdown of RBM3 (**Figure S5**). To evaluate whether reduced RBM3 expression causes endogenous pre-miRNAs to acquire a distinct set of factors, we used sucrose gradient fractionation assays optimized to resolve low molecular weight (MW) pre-miRNPs and complexes containing TRBP and Ago2 (**Figure S6A, B**). As measured by continuous A_260_ RNA readings through the gradients, knockdown of RBM3 altered the profile of low MW RNPs and translation machinery. In fractions 1 – 9 from the early RNP portion, TRBP and Ago2 co-sedimented and their distribution peaked in the same fraction (#6) in controls and RBM3 siRNA conditions (**Figure 5A**). However, the distribution of pre-miRNAs was altered by knockdown of RBM3. Northern blots of pre-let-7g in gradient fractions revealed that precursors accumulating after RBM3 knockdown distribute to complexes of lower MW than TRBP/Ago2-containing complexes (**Figure 5A**, **Figure S6B**). In controls, mature let-7g was also detected, along with decreasing amounts of pre-let-7g, in TRBP/Ago2-containing fractions, suggesting that these are active processing complexes (**Figure 5A**). However, mature let-7g was not detected in TRBP/Ago2-containing fractions under RBM3 knockdown conditions, even though these fractions contained some pre-let-7g and exhibited more Dicer activity towards exogenous pre-let-7g (**Figure 5B**). These data suggest that reduced RBM3 expression results in the association of pre-miRNAs with a lower MW complex of factors that attenuate interactions with Dicer. Alternatively, RBM3 may itself facilitate pre-miRNP association with Dicer, but this would have to be reconciled with the enhanced processing of exogenous pre-miRNAs by RBM3 knockdown cell lysates.

**Figure 6:**
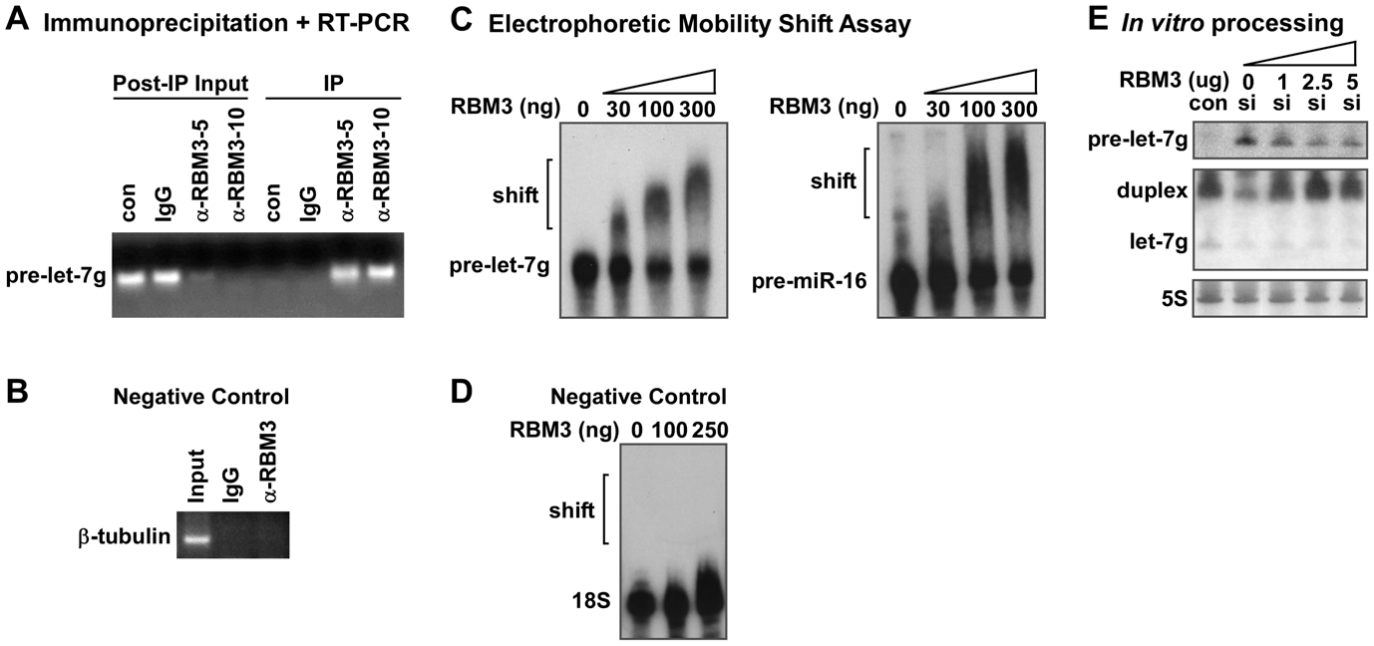
RBM3 associates with pre-miRNAs. (**A**) Gel showing pre-let-7g RT-PCR product amplified from immunoprecipitate (IP) and post-IP input fractions in the following conditions: no treatment (con), IP with pre-immune IgG (IgG), IP with 5 µg and 10 µg of affinity purified anti-RBM3 antibody (α-RBM3-5/10). (**B**) Negative control RT-PCR for β-tubulin. (**C**) Autoradiographs of labeled pre-let-7g and pre-miR-16 resolved on non-denaturing gels after incubation with increasing amounts of purified recombinant RBM3 in electrophoretic mobility shift assays. (**D**) Negative control gel shift using 18S RNA. (**E**) Northern blot showing partial rescue of endogenous pre-let-7g processing in RBM3 knockdown (si) cell lysates by addition of recombinant RBM3 to *in vitro* processing reactions. 5S is loading control.

The effects of RBM3 on pre-miRNA processing may arise from direct interactions between RBM3 and precursors, analogous to what has been shown for LIN28, hnRNPA1, and KSRP [15]–[19]. To test this, we immunoprecipitated RBM3 and probed for pre-let-7g and pre-miR-16 by RT-PCR. Both of these precursors were amplified from the RBM3 immunoprecipitates, but not from IgG control precipitates or from samples in which reverse transcriptase was omitted (**Figure 6A** and **Figure S7**); β-tubulin mRNA was tested as a negative control (**Figure 6B**). In addition, electrophoretic mobility shift assays showed that RBM3 binds pre-let-7g and pre-miR-16 probes directly *in vitro* (**Figure 6C**); 18S RNA was used as a negative control in these assays (**Figure 6D**). Consistent with the idea that RBM3 may regulate pre-miRNP access to Dicer complexes, the processing of endogenous pre-let-7g by cytoplasmic extracts from RBM3-depleted cells was partially rescued by acute addition of recombinant RBM3 (**Figure 6E**). Addition of exogenous RBM3 to extracts from cells in which endogenous RBM3 had been knocked down led to a decrease in pre-let-7g levels, with a concomitant increase of the dsRNA miRNA duplex. These data suggest that RBM3 binds directly to pre-miRNAs to regulate their processing by Dicer.

## Discussion

**Figure 7:**
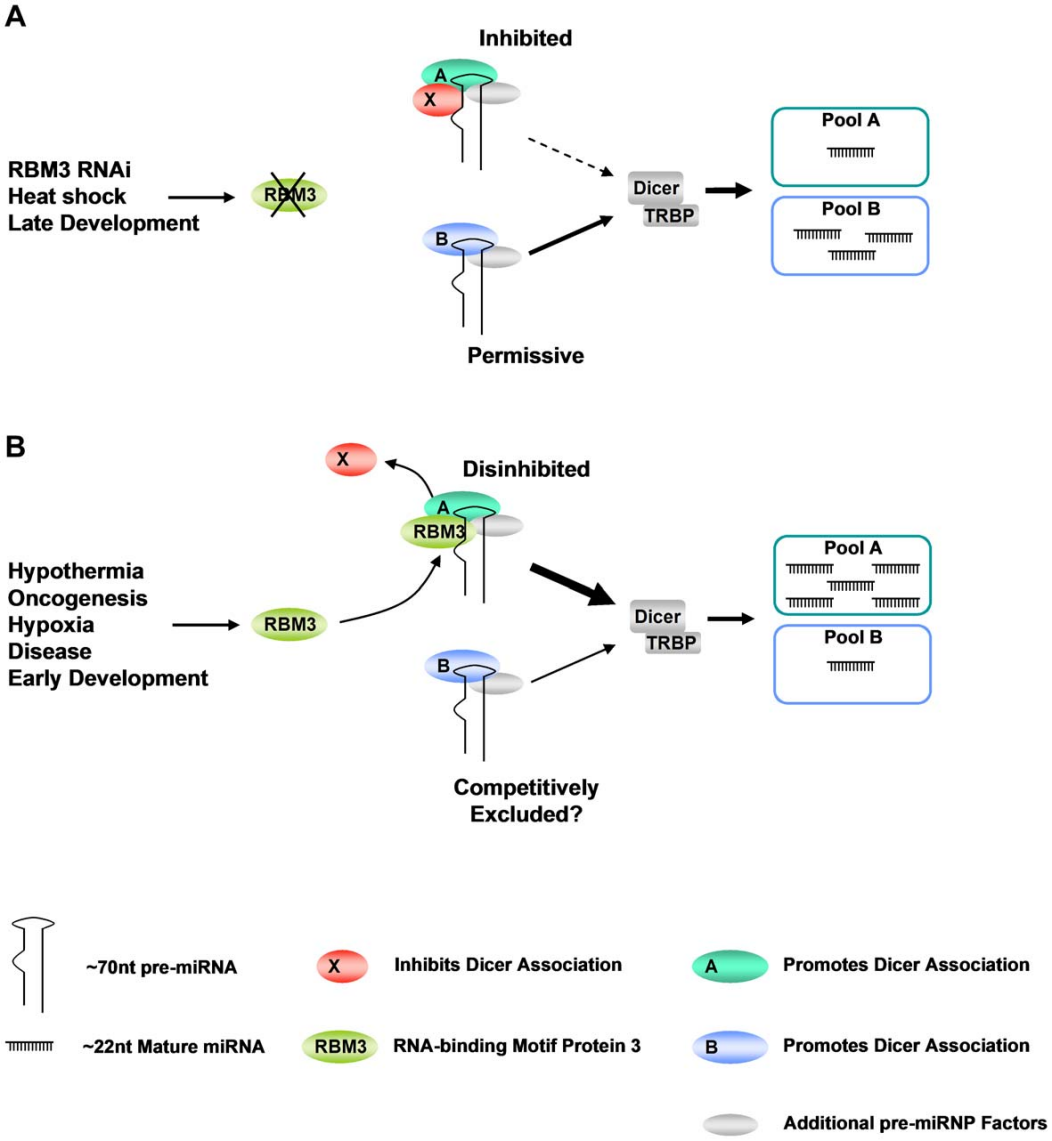
Model of the effects of RBM3 on the posttranscriptional regulation of miRNA biogenesis. (**A**) Pre-miRNAs exist as two or more pools of pre-miRNPs that contain distinct factors (A and B) mediating association with Dicer complexes. One class also binds a factor (X) that inhibits association with Dicer complexes, and is displaceable by RBM3. In the context of low RBM3 levels – such as occurs during RBM3 knockdown, heat shock, and in most cells during later stages of development – miRNA biogenesis is biased towards pool B, which lacks factor “X” and thus has greater access to Dicer. Not shown in this model are the increases in Dicer and Ago2 levels that occur during RBM3 knockdown, which may also facilitate the processing of pre-miRNAs not bound by an inhibitory factor. (**B**) In the context of high RBM3 levels – such as occurs during mild hypothermia, oncogenesis, early development, hypoxia, degenerative disesease, and early development – the inhibitory factor is displaced from pool A by the binding of RBM3 to pre-miRNAs. RBM3 binding thus derepresses pre-miRNAs in pool A and enhances their access to Dicer complexes, and subsequent processing. Access of pre-miRNAs that do not bind RBM3 (pool B) to Dicer is reduced by competitive exclusion.

Our data establish a novel role for RBM3 in the posttranscriptional regulation of miRNA biogenesis, one with important implications for how pre-miRNAs are regulated as RNPs. The majority of miRNAs affected by RBM3 knockdown were downregulated, leading to accumulation of endogenous pre-miRNAs even as Dicer activity remained at or above normal levels against exogenous substrates. We observed that RBM3 associates with pre-miRNAs *in vitro* and *in situ*, suggesting that it is an integral part of larger pre-miRNA ribonucleoprotein complexes (pre-miRNPs). Taken together, these findings suggest that RBM3 regulates the competency of a large proportion of pre-miRNPs to engage catalytically active Dicer complexes. This is consistent with our observation that, in sucrose gradient fractionation assays, knockdown of RBM3 leads to accumulation of pre-miRNAs as lower MW species. A plausible model for these effects is that integration of RBM3 into pre-miRNPs displaces an inhibitory factor from pre-miRNAs that normally attenuates their processing by Dicer (**Figure 7**). Our data favor this de-repression model over one in which RBM3 directly facilitates association of pre-miRNPs with Dicer complexes because exogenous pre-miRNAs are rapidly and efficiently processed by extracts from RBM3 depleted cells. In any case, our results support the broader notion that miRNA biogenesis is differentially regulated by the coordinated effects of many RNA-BPs acting at the level of pre-miRNP formation [3]. The fact that 60% of all miRNAs detected by microarray in B104 cells were downregulated after depletion of RBM3 suggests that RBM3 is a component of most pre-miRNPs. How RBM3 modifies the composition of pre-miRNPs is currently under study.

Our results also bear on the nature of cellular conditions that influence the miRNA pathway via RNA-BPs. Although cold exposure is among the most basic and ubiquitous challenges to living organisms – and an essential aspect of torpor – the cellular responses to this stress are poorly understood. What is clear, however, is that cold exposure can elicit cellular changes that are protective or that limit pathological processes after a physiological insult or trauma [41]. This effect has been exploited clinically in the areas of therapeutic hypothermia during surgery, after spinal chord injury, in traumatic brain injury and stroke, and in organ transplant [41]–[43]. Recent reports indicate that induction of RBM3 is essential for the protective effects of hypothermia in models of perinatal asphyxia [44] and muscle wasting [45]. The present data and our prior work [22], [23] together suggest that a coordinated regulation of miRNA biogenesis and global translation rates by RBM3 are part of the adaptive process to cold exposure. These molecular functions of RBM3 may contribute directly to the protective effects of hypothermia observed in many paradigms. RBM3 is also induced by other physiological stressors [24], and is upregulated in various cancer types [26] and degenerative states [25], [44], potentially coupling these conditions to changes in miRNA expression.

The set of miRNAs regulated by RBM3 includes many with previously established roles in oncogenesis. It has been proposed elsewhere that RBM3 is a protooncogene that blocks apoptosis and enhances proliferation [26]. Moreover, it has been demonstrated that reduction of RBM3 expression – and expression of the highly homologous cold-inducible RNA binding protein (CIRP) – by heat shock impairs the survival of cancer cells and increases their sensitivity to chemotherapeutic compounds [46]. It is reasonable to speculate, then, that the pro-survival functions of RBM3 in cancer cells, and the therapeutic effects of downregulating RBM3 by heat shock, may involve its activity on miRNAs that have previously been linked to oncogenesis (e.g. the miR-17-92 cluster [7], [8]). However, there is also emerging evidence that very high expression of RBM3 within the nuclear compartment in some cancer types is a positive predictor of clinical outcome [47]. This may reflect a different role of nuclear-retained RBM3, or perhaps a change in the expression of key miRNAs (such as those targeting RBM3-regulated checkpoint proteins [47]) at higher RBM3 concentrations.

RBM3 is also dynamically expressed as part of normal physiology during early brain development [27]. In this context, the effects of RBM3 on miRNAs involved in neural differentiation may be significant given that the RNA-BP is highly expressed in multiple brain regions during an early postnatal period, and remains especially high in neural progenitor fields in adulthood [27]. Of particular interest is the finding that RBM3 enhances the biogenesis of all members of the let-7 family, which are involved in neural differentiation [19], [29]. In embryonic stem cells, the processing of let-7 family members is known to be blocked at the Dicer step by LIN28 to maintain pluripotency, and this blockade is negatively regulated by a feedback influence of let-7 and miR-125a [19]. It will be of interest to determine if RBM3 opposes the effects of LIN28 by facilitating negative feedback at an early stage in neural commitment.

Given the range of conditions under which RBM3 is strongly induced, the data we have obtained here concerning its effects on miRNA biogenesis suggest that RBM3 may be a critical regulator of miRNA-dependent events involved in adaptive, developmental and pathophysiological processes. Further mechanistic studies may unveil additional factors that participate with RBM3 in fine tuning miRNA biogenesis responses to particular cellular or developmental conditions. In addition, clinically important insights into the mechanisms of therapeutic hypothermia may emerge from target analyses of RBM3-regulated miRNAs.

## Materials and Methods

### Manipulation of RBM3 levels

Our studies utilized B104 neuroblastoma [48], HeLa and HEK 293T (ATCC) cell lines. Cell lines were transfected with the siRNA (si) duplex listed in **Table S2** at a ratio of 20 nM siRNA per 25 µl RNAiMax to knockdown RBM3, or pcDNA3.1-RBM3 to overexpress the protein using Lipofectamine reagents (Life Technologies); cells were harvested after 48 hrs. For cold-shock (CS) induction of RBM3, B104 cells were placed at 32°C for 24 hours. The B104 cell line was used in miRNA array profiling studies.

### MicroRNA array profiling

The mirVana miRNA isolation kit was used to isolate miRNAs. miRNA array profiling utilized the mouse miRNA microarray from Agilent Technologies (G4472A), which is selective for mature miRNAs; hybridization was done according to their specifications. Microarrays were scanned and analyzed with the Agilent scanner and Genespring software.

The Agilent Feature Extraction tool was used to obtain the signal values and detection above background for the Agilent chip. Processing of the data was performed within the Bioconductor project and the R program software (R is available as Free Software under the terms of the Free Software Foundation's GNU General Public License). The scale normalization method used was proposed by Yang et al. [49], [50] and is further elaborated by Smyth and Speed [51]. Log-values were scaled to have the same median-absolute-deviation (MAD) across arrays. The 578 Human miRNAs were filtered down to 216 by excluding those miRNAs with signals not detected above background for all the samples.

The fold changes and standard errors were estimated by fitting a linear model with allowance for paired samples for each gene and empirical Bayes smoothing was applied to the standard errors for all the samples at the same time. The linear modeling approach and the empirical Bayes statistics as implemented in the Limma package in the R software were employed for differential expression analysis. Statistics were obtained for transcripts with the multiple testing adjusted (Benjamini-Hochberg) p-values level of 0.05. MicroRNA array data are MIAME compliant and have been deposited in the GEO database (accession # pending).

### Northern blotting

Per treatment condition, 10 µg of total RNA was resolved on a 15% TBE-urea gel, transferred to a nylon membrane (250 mA for 1.5 hours), then cross-linked with UV. Radiolabeled RNA probes (10^6^ cpm/ml) were hybridized at 42°C overnight. Membranes were washed and exposed to X-ray film.

### Western blotting

Samples were lysed in a buffer composed of 1% triton X-100, 1 mM Tris-HCl (pH 7.4), 150 mM NaCl, and protease inhibitor cocktail (Roche), then boiled in NuPage SDS Buffer (Life Technologies) and resolved on 4–12% tris-glycine gels for immunoblotting with antibodies against RBM3 (1∶2000, made in house), β-actin (1∶10,000 Sigma), Ago2 (1∶1000 Abnova), Dicer (1∶1000 Abnova), and ribosomal protein S20 (1∶1000, made in house).

### Sucrose gradient centrifugation analysis of pre-miRNPs and translation machinery

A total of 2×10^7^ B104 cells were lysed with ice-cold buffer A: 20 mM Tris, pH 7.4, 100 mM KCl, 10 mM MgCl_2_, 0.5 mg/ml heparin, 2 mM DTT, protease inhibitor cocktail (Roche), 100 µg/ml cycloheximide (Sigma), and 0.3% Igepal-600 (Sigma). Cytoplasmic extracts were obtained after centrifugation at 30,000 x g for 15 min at 4°C, loaded onto a linear gradients of 10–50% (to broadly resolve translation machinery) or 15–55% (for finer resolution of pre-miRNP complexes) sucrose in buffer A, and centrifuged at 100,000 x g for 3 h, or 30,000 x g for 18 h (4°C), respectively. A continuous reading of RNA distribution through the gradients was obtained by upward displacement of the sucrose with purdenz (Accurate Chemical & Scientific Corporation) through an ISCO UA-6 UV monitor reading absorbance at 260 nm. Half milliliter fractions were collected through the entire (11 mL) gradients and analyzed by Northern and Western blotting. Protein was precipitation by the TCA/acetone method and RNA was isolated from sucrose gradient fractions with Trizol LS according to manufacturer protocol (Life Technologies).

### *In Situ* Hybridization

B104 cells grown on glass coverslips were fixed with 4% paraformaldehyde, and *in situ* hybridization was performed as previously described [52] with 20 ng of 5′-DIG-labeled LNA hybridization probes complementary to mouse mature let-7g and pre-let-7g (Exiqon). Fluorescent images were collected on a Zeiss LSM 710 laser scanning confocal microscope (LSCM).

### Electrophoretic Mobility Shift Assay

A ^32^P-labeled precursor transcript (125 fmol of either pre-let-7g, pre-miR-16, or 18S RNA) was incubated alone or with increasing concentrations of rRBM3 (ranging from 30 ng to 300 ng) in the following buffer: 5 mM Tris (pH 7.4), 12.5 mM NaCl, 0.05 mM EDTA, 0.05 mg/ml tRNA, 0.05 mg/ml heparin and 0.01% Igepal in a total volume of 10 µl. The reactions were incubated at 4°C for 30 min, and then 2.8 µl of RNA loading dye was added to each reaction and separated on a 5% native Tris-glycine polyacrylamide gel.

### Preparation and purification of RNA probes

Northern probes were *in vitro* transcribed and labeled with [γ-^32^P] UTP according to the mirVana miRNA Probe Construction kit protocol (Life Technologies) and purified on 15% TBE-acrylamide gels. The gel shift probes were prepared similarly with the T7 MegaShortscript kit (Life Technologies). All ^32^P-labeled RNA transcripts were purified on 15% TBE-acrylamide gels. The synthetic pre-let-7g transcript used in processing assays was prepared cold with T7 MegaShortscript (Life Technologies) for 5′-labeling. To prepare endogenous precursors for processing assays, total isolated RNA was run on a 15% TBE-polyacrylamide gel and bands corresponding to oligonucleotides of 50–100 nt in length were excised and purified from the gel. Elution was carried out overnight with Probe elution buffer (Life Technologies). The eluate was then precipitated using 3 volumes of 100% ethanol. In end-labeling experiments, the 5′ phosphate groups were removed by using alkaline phosphatase (New England Biolabs) for 1h at 37°C, followed by phenol/choloroform purification and ethanol precipitation. The purified RNAs were 5′ end-labeled with [γ-^32^P] ATP by using polynucleotide kinase (New England Biolabs). Following labeling, RNAs were once again resolved on 15% TBE-polyacrylamide gels and the major bands ∼70 nt and ∼100 nt were excised and gel purified. The biotinylated pre-let-7g and pre-mir-16 used in pulldown experiments were transcribed with the T7 MegaShortscript (Life Technologies) with the addition of 7.5 mM Biotin-AG (Thermoscientific) to the transcription mix, followed by column purification. Oligonucleotides used for the T7 RNA polymerase reactions are shown in **Table S2**.

### Subcellular RNA fractionation

Subcellular fractionation and extraction of RNA was performed with the mirVana PARIS kit (Life Technologies), followed by Northern blot analysis.

### Pre-miRNA processing assays

Processing assays were performed as described previously [53] with a few alterations. Cell extracts were lysed in 30 mM Hepes, pH 7.4, 100 mM KCl, 5 mM MgCl_2_, 10% glycerol, 0.5 mM DTT, 0.1 U/µl RNase OUT (Life Technologies), and protease inhibitors (Roche). Lysed samples were sonicated and centrifuged at 4°C, 13,000 rpm. Dicer-complex enriched sucrose gradient fractions were concentrated with Amicon Ultra 10K centrifugal filters (Millipore), and the final protein concentration determined by Bradford (Bio-Rad). A volume of the resulting concentrates containing 10 µg of protein was adjusted such that the following buffer components were present at the indicated final concentrations: 5 mM ATP, 7.5 mM MgCl_2_, 0.5 U RNase OUT (Life Technologies), 0.25×10^5^ cpm of the labeled synthetic precursor. The reaction was set up on ice and incubated for 45 min at 37°C. Samples were boiled for 1 min at 95°C and loaded on a 15% denaturing TBE-urea polyacrylamide gel (Life Technologies) and exposed to X-ray film. The same final buffer and reaction conditions were used in assays of the processing of labeled endogenous pre-miRNAs by B104 cytoplasmic extracts. For processing assays utilizing total cell extracts, lysates were prepared as described above and readjusted to 10 mg/ml.

### Immunoprecipitation of RBM3-miRNA-containing complexes and RT-PCR

Cell extracts were prepared according to the Magna-RIP RNA-binding protein immunoprecipitation kit protocol (Millipore). Samples were immunoprecipitated with 5 µg of anti-Rabbit IgG as a control or 5–10 µg of anti-RBM3 antibody at 4°C overnight. RNA was purified according to the manufacturer protocol. Semi-quantitative PCR was performed to detect precipitated precursor miRNAs using SuperScript III One-Step RT-PCR System (Life Technologies) with primer sets for pre-let-7g, pre-miR-16, and β-tubulin (ValueGene; see **Table S2**).

### Biotin-pre-miRNA pull-down

Biotinylated pre-let-7g pull-down was performed as previously described [14] with a few modifications. Streptavadin Dynabeads (Life Technologies) were washed according to the manufacturer's protocol, then blocked with 0.1 mg/ml tRNA for 30 min at room temperature. The beads were then incubated with biotinylated pre–let-7g (500 nM) for 15 min, followed by washing. Cell extracts were lysed in 20 mM Tris pH 7.4, 200 mM NaCl, 2.5 mM MgCl_2_, 0.05% NP40, 60 U/ml RNase OUT (Life Technologies), 1 mM DTT, and protease inhibitors (Roche), and added to the beads for overnight incubation at 4°C. The beads were washed three times in lysis buffer and the bound proteins were analyzed by Western blotting with antibodies to Dicer (Abnova, 1∶500), Ago2 (Abnova, 1∶1000), and TRBP (Abfrontier, 1∶1000).

### Quantitative RT-PCR

Levels of pre-let-7g in RNA samples from B104 cells under control and RBM3 knockdown conditions were measured by quantitative RT-PCR using the GenoExplorer microRNA qRT-PCR Kit from GenoSensor Corporation (Tempe, AZ). Three biological replicates were run per condition, with 4 technical replicates per sample. Ligation and extension of RNAs and real-time amplification of pre-let-7g and U6 by qPCR with specific and universal primers were conducted according to the manufacturers recommendations using their primer sets. The relative levels of pre-let-7g in control and RBM3 knockdown samples were calculated using the ΔΔCt method with U6 as the reference.

### Quantification of Western blots, Northern blots and Autoradiograms

Quantification of Northern blot, Western blot, and processing autoradiograms utilized the Alpha Ease software. Group data compiled in Excel files and subsequently analyzed for statistical significance in Prizm (GraphPad).

## Supporting Information

Figure S1 **Knockdown and overexpression of RBM3 in the B104 neuronal cell line.** (**A**) Western blot showing RBM3 levels 36 hours after transfection with no siRNA (con), a scrambled siRNA (scr), a siRNA targeting RBM3 (RBM3), or an expression construct containing the RBM3 open reading frame downstream of a CMV promoter in the pcDNA3.1 vector (o/x). β-actin served as a loading control. Mock transfections were used throughout the study as introduction of scrambled siRNA induced slight elevations in RBM3. (**B**) Northern blots showing pre-let-7g and mature let-7g (and 5S loading control) in samples from the same treatments shown in panel a. Expression of mature let-7g was impaired by knockdown of RBM3 and enhanced by overexpression of RBM3. A slightly elevated level of let-7g was present in the scrambled siRNA sample, consistent with a slight elevation of RBM3 levels. (**C**) Western blots of RBM3 in triplicate control, knockdown (siRNA) and overexpression experiments used for microarray profiling of miRNA expression, and as part of subsequent Northern blot validation studies. RBM3 levels were reliably reduced by over 90% by siRNA, and overexpressed with a CMV promoter-based construct at levels mimicking induction after cold-shock.
(PDF)

Figure S2 **Bidirectional modulation of miRNA expression by manipulation of RBM3.** (**A**) Upper panels: Northern blots showing pre-miR-125a and mature miR-125a-5p (and 5S loading control) in samples from B104 cells under control (con), RBM3 knockdown (si), and RBM3 overexpression (o/x). Lower panels: Northern blots showing pre-let-7i and mature let-7i in the same samples. (**B**) Northern blot for pre-miR-125a using a probe complementary to the full sequence; 5S is the loading control.
(PDF)

Figure S3 **Cold-shock induction of RBM3 recapitulates the effects of RBM3 overexpression on miRNA expression.** (**A** & **B**) Northern blots showing levels of precursor and mature forms of let-7g (**A**) and miR-30b (**B**) along with 5S RNA (loading control) in B104 cells under the following conditions: control (con), RBM3 knockdown (siRNA), RBM3 overexpression (o/x), and cold-shock (32°C for 24 hrs). Lower panels in (**A**) show that the manipulations of RBM3 do not alter levels of tRNAs as visualized by Northern blotting for tRNA-Lys and ethidium bromide staining of the corresponding gel. (**C**) Full Northern blot of let-7 from Figure 2 of the main text demonstrating that the enhancement of mature let-7g biogenesis by cold shock requires RBM3 induction. Relative to cells maintained at 37°C, let-7g is elevated under conditions of mild hypothermia in control B104 cells, but not in cells transfected with RBM3 siRNA. 5S RNA is the loading control. Western blots of RBM3 (lower panels) show induction at 32°C in controls, but greatly attenuated expression at both temperatures in the siRNA condition. β-actin is the loading control.
(PDF)

Figure S4 **RBM3 regulates levels of Dicer complex components in HeLa cells.** (**A** & **B**) Western blots showing the relative abundance of Dicer, TRBP (**A**), and Ago2 (**B**) in Hela cells after knockdown (si) and overexpression (o/x) of RBM3, relative to control (con). (**C**) Western blot showing RBM3 expression after knockdown and overexpression of a myc-tagged version of RBM3. β-actin was used as a loading control.
(PDF)

Figure S5 **Components of the miRNA processing machinery are still able to assemble onto exogenous pre-miRNA after knockdown of RBM3.** Lysates of B104 cells maintained under control conditions (left panels) or transfected with a siRNA to RBM3 (right panels) were incubated with biotinylated pre-let-7i or pre-miR-16, followed by retrieval of bound complexes using streptavadin Dynabeads. Incubation with beads alone (beads) was used to control for non-specific associations. Dicer, TRBP, and Ago2 were retrieved in larger amounts from RBM3 siRNA-treated cells than from controls, consistent with elevated levels of these factors in the RBM3 knockdown condition.
(PDF)

Figure S6 **Knockdown of RBM3 alters the formation of monosomes and polysomes, and the relative fractionation of pre-miRNPs relative to miRNA processing factors.** (**A**) RNA-containing complexes in lysates from B104 cells maintained under control conditions (left panels) or transfected with RBM3 siRNA (right panels) were resolved by centrifugation through a 15%-55% linear sucrose gradient. The traces show continuous A260 readings through the gradients; the top of the gradient is at the left of each trace. The positions of 40S and 60S ribosomal subunits and 80S monosomes are indicated. Western blots showing the distribution of the small ribosomal subunit protein S20 across all fractions collected from the gradient (22×0.5 mL fractions). The solid line insets delineate the set of complexes resolved by higher resolution gradients. These are shown in Figure S6B and include pre-miRNA-ribonucleoprotein complexes. (**B**) Optimization of the fractionation parameters to resolve lower molecular (MW) weight ribonucleoprotein particles (RNPs) reveals an altered fractionation of miRNA precursor-containing RNPs (pre-miRNPs). Traces of A260 through the 22 fraction (11 mL) gradients show that a set of low MW RNPs is altered in RBM3 siRNA-transfected (right panels) vs control (left panels) B104 cells. Major low MW RNP peaks are labeled by the fraction they correspond to; 40S and 60S ribosomal subunits are indicated for reference. Northern blots presented in Figure 4 of the main text are shown below each gradient trace here to show the distribution and levels of pre-let-7g and mature let-7g in the first 9 fractions of each gradient relative to the A260 trace; fraction 6 (red) contains the peak distribution of TRBP and Ago2.
(PDF)

Figure S7 **Immunoprecipitation of miRNA precursors with RBM3.** (**A**) Gel showing RT-PCR products amplified input and immunoprecipitate (IP) fractions with primers specific for pre-miR-16; affinity purified α-RBM3 polyclonal and a pre-immune IgG control were used in immunoprecipitation reactions. The results are similar to those presented for pre-let-7g in Figure 4 of the main text. (**B**) Gel showing the effects of omitting reverse transcriptase (RT) from PCR reactions used to detect pre-let-7g in α-RBM3 immunoprecipitate and input fractions. The pre-let-7g product is only amplified in each fraction if RT is included.
(PDF)

Table S1 **Agilent miRNA array data.** Table listing 159 miRNAs showing significant expression changes, out of 216 detected above threshold using Agilent miRNA microarrays, after knockdown of RBM3 in the B104 neuronal cell line.
(PDF)

Table S2 **Oligonucleotides used.** Table listing oligonucleotides used in Northern blot, *in situ* hybridization, RT-PCR, gel shift, processing, and pull-down assays, and siRNAs used to knockdown RBM3.
(PDF)

## References

[1] Filipowicz W, Jaskiewicz L, Kolb FA, Pillai RS (2005). Post-transcriptional gene silencing by siRNAs and miRNAs.. Curr Opin Struct Biol.

[2] Vasudevan S, Tong Y, Steitz JA (2007). Switching from repression to activation: microRNAs can up-regulate translation.. Science.

[3] Siomi H, Siomi MC (2010). Posttranscriptional regulation of microRNA biogenesis in animals.. Mol Cell.

[4] Houbaviy HB, Murray MF, Sharp PA (2003). Embryonic stem cell-specific MicroRNAs.. Dev Cell.

[5] Schratt GM, Tuebing F, Nigh EA, Kane CG, Sabatini ME (2006). A brain-specific microRNA regulates dendritic spine development.. Nature.

[6] Cheng HY, Papp JW, Varlamova O, Dziema H, Russell B (2007). microRNA modulation of circadian-clock period and entrainment.. Neuron.

[7] He L, Thomson JM, Hemann MT, Hernando-Monge E, Mu D (2005). A microRNA polycistron as a potential human oncogene.. Nature.

[8] Dews M, Homayouni A, Yu D, Murphy D, Sevignani C (2006). Augmentation of tumor angiogenesis by a Myc-activated microRNA cluster.. Nat Genet.

[9] Gao FB (2010). Context-dependent functions of specific microRNAs in neuronal development.. Neural Dev.

[10] O'Connell RM, Rao DS, Chaudhuri AA, Baltimore D (2010). Physiological and pathological roles for microRNAs in the immune system.. Nat Rev Immunol.

[11] Bartel DP (2004). MicroRNAs: genomics, biogenesis, mechanism, and function.. Cell.

[12] Liu J, Carmell MA, Rivas FV, Marsden CG, Thomson JM (2004). Argonaute2 is the catalytic engine of mammalian RNAi.. Science.

[13] Gregory RI, Chendrimada TP, Cooch N, Shiekhattar R (2005). Human RISC couples microRNA biogenesis and posttranscriptional gene silencing.. Cell.

[14] Kedde M, Strasser MJ, Boldajipour B, Oude Vrielink JA, Slanchev K (2007). RNA-binding protein Dnd1 inhibits microRNA access to target mRNA.. Cell.

[15] Viswanathan SR, Daley GQ, Gregory RI (2008). Selective blockade of microRNA processing by Lin28.. Science.

[16] Guil S, Caceres JF (2007). The multifunctional RNA-binding protein hnRNP A1 is required for processing of miR-18a.. Nat Struct Mol Biol.

[17] Trabucchi M, Briata P, Garcia-Mayoral M, Haase AD, Filipowicz W (2009). The RNA-binding protein KSRP promotes the biogenesis of a subset of microRNAs.. Nature.

[18] Michlewski G, Caceres JF (2010). Antagonistic role of hnRNP A1 and KSRP in the regulation of let-7a biogenesis.. Nat Struct Mol Biol.

[19] Rybak A, Fuchs H, Smirnova L, Brandt C, Pohl EE (2008). A feedback loop comprising lin-28 and let-7 controls pre-let-7 maturation during neural stem-cell commitment.. Nat Cell Biol.

[20] Danno S, Nishiyama H, Higashitsuji H, Yokoi H, Xue JH (1997). Increased transcript level of RBM3, a member of the glycine-rich RNA-binding protein family, in human cells in response to cold stress.. Biochem Biophys Res Commun.

[21] Williams DR, Epperson LE, Li W, Hughes MA, Taylor R (2005). Seasonally hibernating phenotype assessed through transcript screening.. Physiol Genomics.

[22] Dresios J, Aschrafi A, Owens GC, Vanderklish PW, Edelman GM (2005). Cold stress-induced protein Rbm3 binds 60S ribosomal subunits, alters microRNA levels, and enhances global protein synthesis.. Proc Natl Acad Sci U S A.

[23] Smart F, Aschrafi A, Atkins A, Owens GC, Pilotte J (2007). Two isoforms of the cold-inducible mRNA-binding protein RBM3 localize to dendrites and promote translation.. J Neurochem.

[24] Wellmann S, Buhrer C, Moderegger E, Zelmer A, Kirschner R (2004). Oxygen-regulated expression of the RNA-binding proteins RBM3 and CIRP by a HIF-1-independent mechanism.. J Cell Sci.

[25] Kita H, Carmichael J, Swartz J, Muro S, Wyttenbach A (2002). Modulation of polyglutamine-induced cell death by genes identified by expression profiling.. Hum Mol Genet.

[26] Sureban SM, Ramalingam S, Natarajan G, May R, Subramaniam D (2008). Translation regulatory factor RBM3 is a proto-oncogene that prevents mitotic catastrophe.. Oncogene.

[27] Pilotte J, Cunningham BA, Edelman GM, Vanderklish PW (2009). Developmentally regulated expression of the cold-inducible RNA-binding motif protein 3 in euthermic rat brain.. Brain Res.

[28] Shibata M, Nakao H, Kiyonari H, Abe T, Aizawa S (2011). MicroRNA-9 regulates neurogenesis in mouse telencephalon by targeting multiple transcription factors.. J Neurosci Res.

[29] Wulczyn FG, Smirnova L, Rybak A, Brandt C, Kwidzinski E (2007). Post-transcriptional regulation of the let-7 microRNA during neural cell specification.. FASEB J.

[30] Gao J, Wang WY, Mao YW, Graff J, Guan JS (2010). A novel pathway regulates memory and plasticity via SIRT1 and miR-134.. Nature.

[31] Kole AJ, Swahari V, Hammond SM, Deshmukh M (2011). miR-29b is activated during neuronal maturation and targets BH3-only genes to restrict apoptosis.. Genes Dev.

[32] Ruby JG, Jan CH, Bartel DP (2007). Intronic microRNA precursors that bypass Drosha processing.. Nature.

[33] Okamura K, Hagen JW, Duan H, Tyler DM, Lai EC (2007). The mirtron pathway generates microRNA-class regulatory RNAs in Drosophila.. Cell.

[34] Grishok A, Pasquinelli AE, Conte D, Li N, Parrish S (2001). Genes and mechanisms related to RNA interference regulate expression of the small temporal RNAs that control C. elegans developmental timing.. Cell.

[35] Politz JC, Hogan EM, Pederson T (2009). MicroRNAs with a nucleolar location.. RNA.

[36] Haase AD, Jaskiewicz L, Zhang H, Laine S, Sack R (2005). TRBP, a regulator of cellular PKR and HIV-1 virus expression, interacts with Dicer and functions in RNA silencing.. EMBO Rep.

[37] Forman JJ, Legesse-Miller A, Coller HA (2008). A search for conserved sequences in coding regions reveals that the let-7 microRNA targets Dicer within its coding sequence.. Proc Natl Acad Sci U S A.

[38] Johnson SM, Grosshans H, Shingara J, Byrom M, Jarvis R (2005). RAS is regulated by the let-7 microRNA family.. Cell.

[39] Heo I, Joo C, Kim YK, Ha M, Yoon MJ (2009). TUT4 in concert with Lin28 suppresses microRNA biogenesis through pre-microRNA uridylation.. Cell.

[40] Hagan JP, Piskounova E, Gregory RI (2009). Lin28 recruits the TUTase Zcchc11 to inhibit let-7 maturation in mouse embryonic stem cells.. Nat Struct Mol Biol.

[41] Lampe JW, Becker LB (2011). State of the art in therapeutic hypothermia.. Annu Rev Med.

[42] Dietrich WD (2009). Therapeutic hypothermia for spinal cord injury.. Crit Care Med.

[43] Dietrich WD, Atkins CM, Bramlett HM (2009). Protection in animal models of brain and spinal cord injury with mild to moderate hypothermia.. J Neurotrauma.

[44] Chip S, Zelmer A, Ogunshola OO, Felderhoff-Mueser U, Nitsch C (2011). The RNA-binding protein RBM3 is involved in hypothermia induced neuroprotection.. Neurobiol Dis.

[45] Ferry AL, Vanderklish PW, Dupont-Versteegden EE (2011). Enhanced survival of skeletal muscle myoblasts in response to overexpression of cold shock protein, RBM3.. American J Cell Phys.

[46] Zeng Y, Kulkarni P, Inoue T, Getzenberg RH (2009). Down-regulating cold shock protein genes impairs cancer cell survival and enhances chemosensitivity.. J Cell Biochem.

[47] Ehlēn O, Nodin B, Rexhepaj E, Bråndstedt J, Uhlen M (2011). RBM3-regulated genes promote DNA integrity and affect clinical outcome in epithelial ovarian cancer.. Translational Oncol.

[48] Schubert D, Heinemann S, Carlisle W, Tarikas H, Kimes B (1974). Clonal cell lines from the rat central nervous system.. Nature.

[49] Yang YH, Dudoit S, Luu P, Lin DM, Peng V (2002). Normalization for cDNA microarray data: a robust composite method addressing single and multiple slide systematic variation.. Nucleic Acids Res.

[50] Yang YH, Dudoit S, Luu P, Speed TP, Bittner MLYC, Dorsel AN, Dougherty ER (2001). Normalization for cDNA microarray data.. Microarrays: Optical Technologies and Informatics.

[51] Smyth GK, Speed TP, Carter D (2003). Normalization of cDNA microarray data.. METHODS: Selecting Candidate Genes from DNA Array Screens: Application to Neuroscience.

[52] Politz JC, Zhang F, Pederson T (2006). MicroRNA-206 colocalizes with ribosome-rich regions in both the nucleolus and cytoplasm of rat myogenic cells.. Proc Natl Acad Sci U S A.

[53] Leuschner PJ, Martinez J (2007). In vitro analysis of microRNA processing using recombinant Dicer and cytoplasmic extracts of HeLa cells.. Methods.

