# Supplementary figures and images for: Widespread Regulation of miRNA Biogenesis at the Dicer Step by the Cold-Inducible RNA-Binding Protein, RBM3
Source: PLoS One. 2011 Dec 1;6(12):e28446. doi: 10.1371/journal.pone.0028446 (PMC3228759; doi:10.1371/journal.pone.0028446)

Figure S1

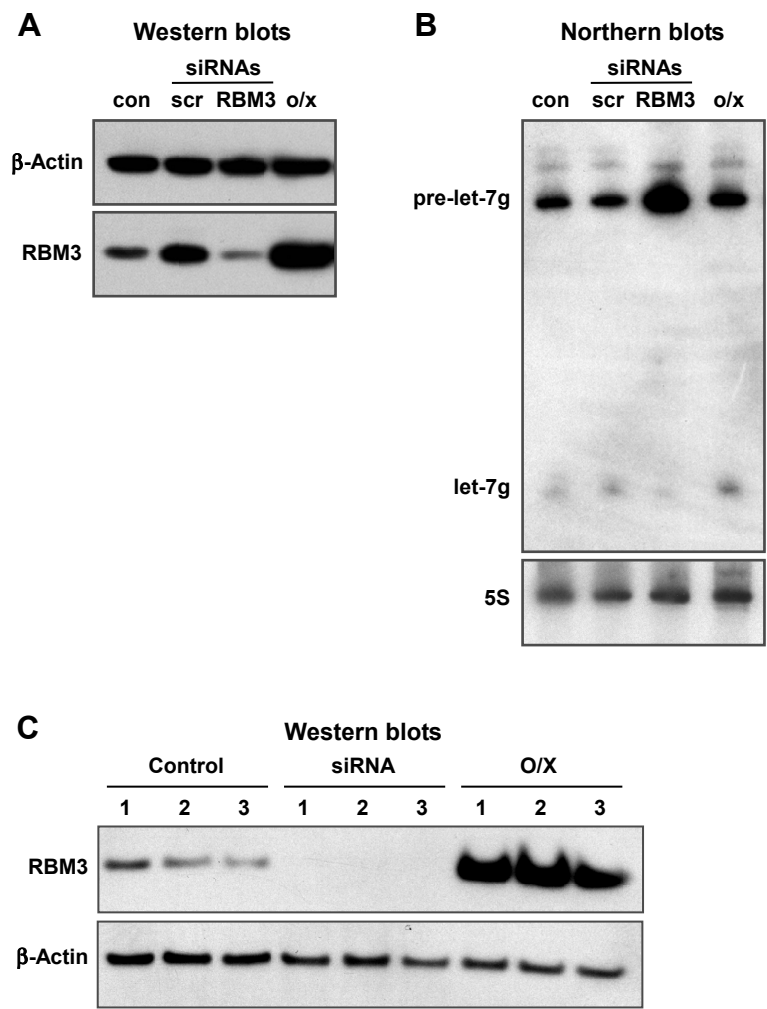

Supplement: Figure S1 — Knockdown and overexpression of RBM3 in the B104 neuronal cell line. (A) Western blot showing RBM3 levels 36 hours after transfection with no siRNA (con), a scrambled siRNA (scr), a siRNA targeting RBM3 (RBM3), or an expression construct containing the RBM3 open reading frame downstream of a CMV promoter in the pcDNA3.1 vector (o/x). β-actin served as a loading control. Mock transfections were used throughout the study as introduction of scrambled siRNA induced slight elevations in RBM3. (B) Northern blots showing pre-let-7g and mature let-7g (and 5S loading control) in samples from the same treatments shown in panel a. Expression of mature let-7g was impaired by knockdown of RBM3 and enhanced by overexpression of RBM3. A slightly elevated level of let-7g was present in the scrambled siRNA sample, consistent with a slight elevation of RBM3 levels. (C) Western blots of RBM3 in triplicate control, knockdown (siRNA) and overexpression experiments used for microarray profiling of miRNA expression, and as part of subsequent Northern blot validation studies. RBM3 levels were reliably reduced by over 90% by siRNA, and overexpressed with a CMV promoter-based construct at levels mimicking induction after cold-shock. (PDF) [file pone.0028446.s001.pdf]

Figure S2

**A**

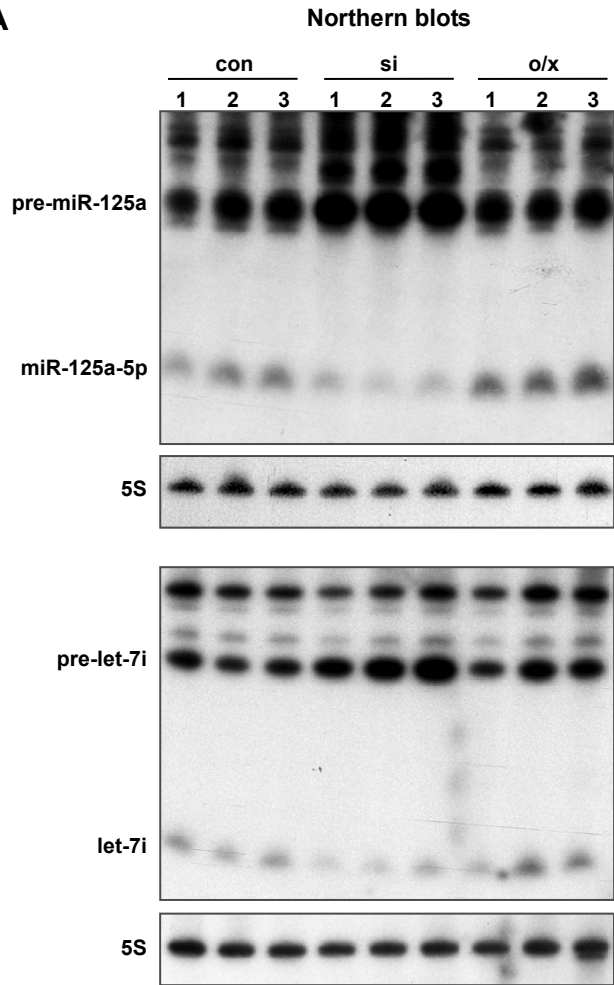

**B**

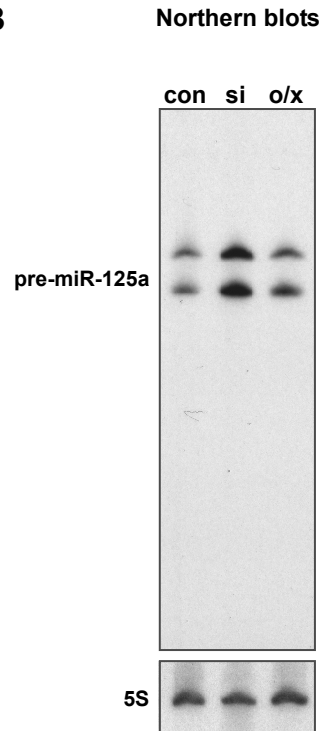

Supplement: Figure S2 — Bidirectional modulation of miRNA expression by manipulation of RBM3. (A) Upper panels: Northern blots showing pre-miR-125a and mature miR-125a-5p (and 5S loading control) in samples from B104 cells under control (con), RBM3 knockdown (si), and RBM3 overexpression (o/x). Lower panels: Northern blots showing pre-let-7i and mature let-7i in the same samples. (B) Northern blot for pre-miR-125a using a probe complementary to the full sequence; 5S is the loading control. (PDF) [file pone.0028446.s002.pdf]

Figure S3

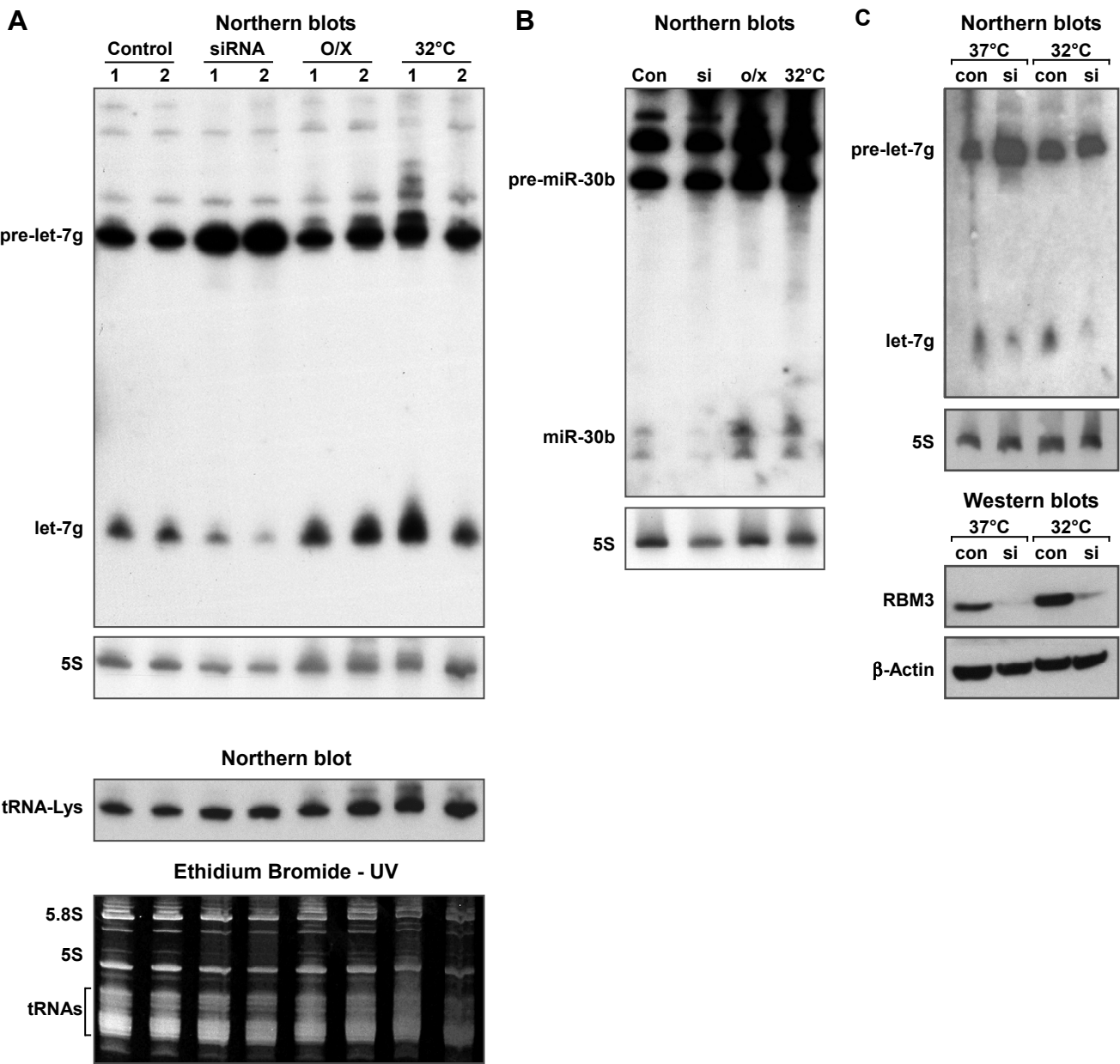

Supplement: Figure S3 — Cold-shock induction of RBM3 recapitulates the effects of RBM3 overexpression on miRNA expression. (A & B) Northern blots showing levels of precursor and mature forms of let-7g (A) and miR-30b (B) along with 5S RNA (loading control) in B104 cells under the following conditions: control (con), RBM3 knockdown (siRNA), RBM3 overexpression (o/x), and cold-shock (32°C for 24 hrs). Lower panels in (A) show that the manipulations of RBM3 do not alter levels of tRNAs as visualized by Northern blotting for tRNA-Lys and ethidium bromide staining of the corresponding gel. (C) Full Northern blot of let-7 from Figure 2 of the main text demonstrating that the enhancement of mature let-7g biogenesis by cold shock requires RBM3 induction. Relative to cells maintained at 37°C, let-7g is elevated under conditions of mild hypothermia in control B104 cells, but not in cells transfected with RBM3 siRNA. 5S RNA is the loading control. Western blots of RBM3 (lower panels) show induction at 32°C in controls, but greatly attenuated expression at both temperatures in the siRNA condition. β-actin is the loading control. (PDF) [file pone.0028446.s003.pdf]

Figure S4

HeLa cells

**A**

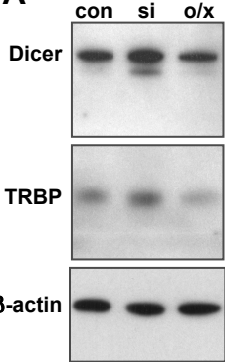

**B**

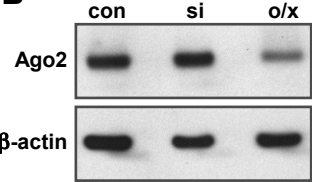

**C**

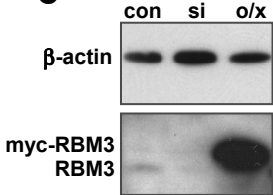

Supplement: Figure S4 — RBM3 regulates levels of Dicer complex components in HeLa cells. (A & B) Western blots showing the relative abundance of Dicer, TRBP (A), and Ago2 (B) in Hela cells after knockdown (si) and overexpression (o/x) of RBM3, relative to control (con). (C) Western blot showing RBM3 expression after knockdown and overexpression of a myc-tagged version of RBM3. β-actin was used as a loading control. (PDF) [file pone.0028446.s004.pdf]

Figure S5

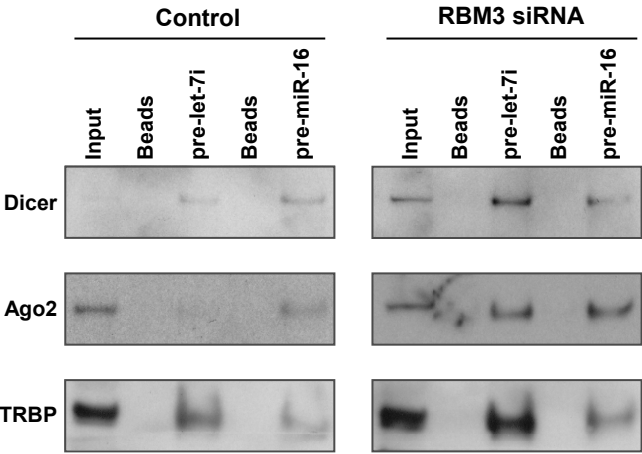

Supplement: Figure S5 — Components of the miRNA processing machinery are still able to assemble onto exogenous pre-miRNA after knockdown of RBM3. Lysates of B104 cells maintained under control conditions (left panels) or transfected with a siRNA to RBM3 (right panels) were incubated with biotinylated pre-let-7i or pre-miR-16, followed by retrieval of bound complexes using streptavadin Dynabeads. Incubation with beads alone (beads) was used to control for non-specific associations. Dicer, TRBP, and Ago2 were retrieved in larger amounts from RBM3 siRNA-treated cells than from controls, consistent with elevated levels of these factors in the RBM3 knockdown condition. (PDF) [file pone.0028446.s005.pdf]

Figure S6

A

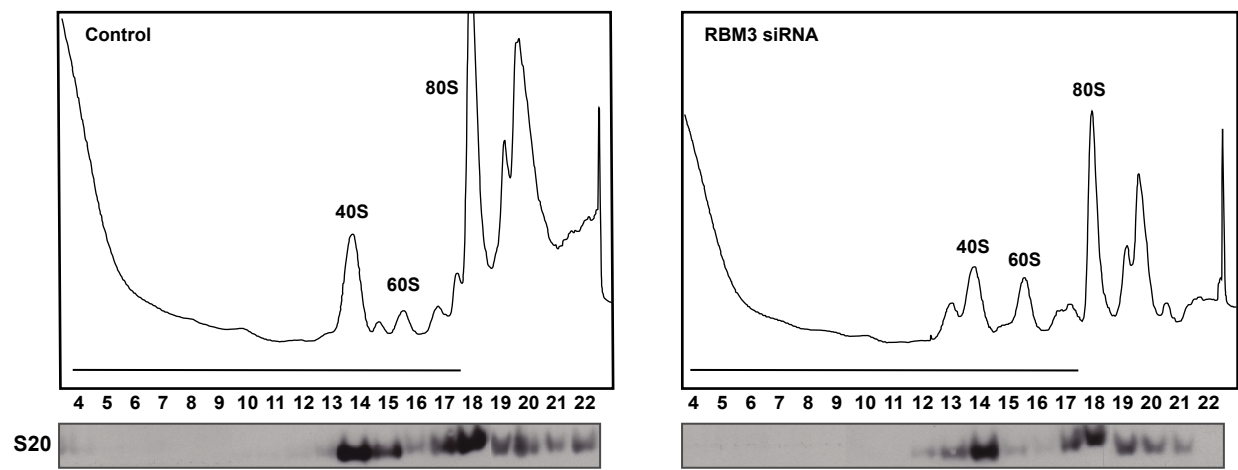

Figure S6 (continued)

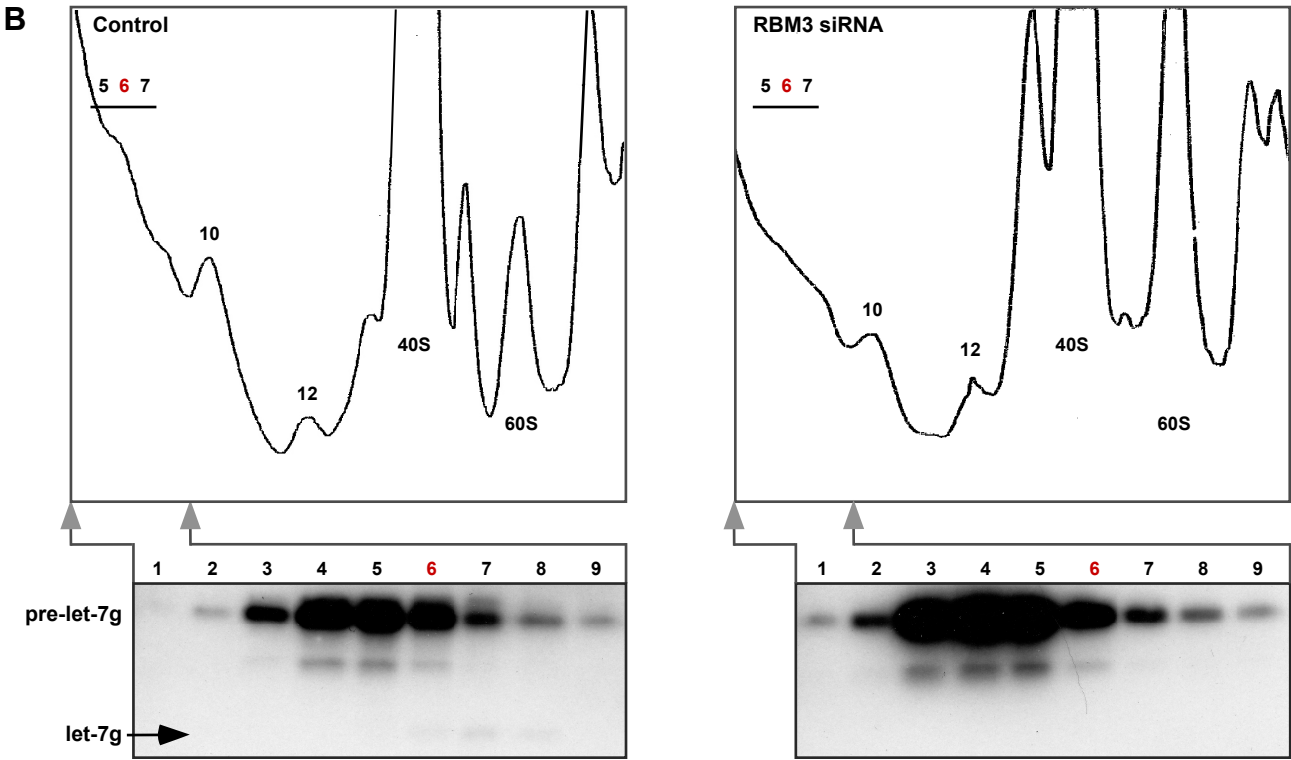

Supplement: Figure S6 — Knockdown of RBM3 alters the formation of monosomes and polysomes, and the relative fractionation of pre-miRNPs relative to miRNA processing factors. (A) RNA-containing complexes in lysates from B104 cells maintained under control conditions (left panels) or transfected with RBM3 siRNA (right panels) were resolved by centrifugation through a 15%-55% linear sucrose gradient. The traces show continuous A260 readings through the gradients; the top of the gradient is at the left of each trace. The positions of 40S and 60S ribosomal subunits and 80S monosomes are indicated. Western blots showing the distribution of the small ribosomal subunit protein S20 across all fractions collected from the gradient (22×0.5 mL fractions). The solid line insets delineate the set of complexes resolved by higher resolution gradients. These are shown in Figure S6B and include pre-miRNA-ribonucleoprotein complexes. (B) Optimization of the fractionation parameters to resolve lower molecular (MW) weight ribonucleoprotein particles (RNPs) reveals an altered fractionation of miRNA precursor-containing RNPs (pre-miRNPs). Traces of A260 through the 22 fraction (11 mL) gradients show that a set of low MW RNPs is altered in RBM3 siRNA-transfected (right panels) vs control (left panels) B104 cells. Major low MW RNP peaks are labeled by the fraction they correspond to; 40S and 60S ribosomal subunits are indicated for reference. Northern blots presented in Figure 4 of the main text are shown below each gradient trace here to show the distribution and levels of pre-let-7g and mature let-7g in the first 9 fractions of each gradient relative to the A260 trace; fraction 6 (red) contains the peak distribution of TRBP and Ago2. (PDF) [file pone.0028446.s006.pdf]

Figure S7

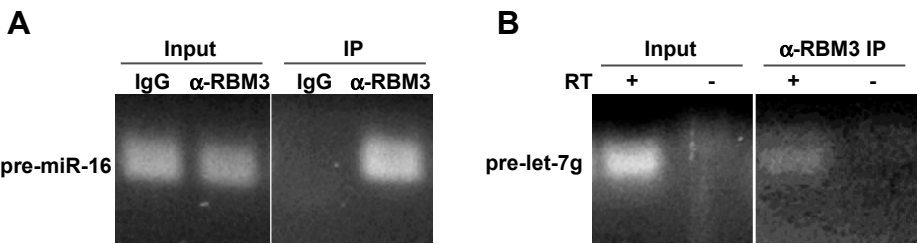

Supplement: Figure S7 — Immunoprecipitation of miRNA precursors with RBM3. (A) Gel showing RT-PCR products amplified input and immunoprecipitate (IP) fractions with primers specific for pre-miR-16; affinity purified α-RBM3 polyclonal and a pre-immune IgG control were used in immunoprecipitation reactions. The results are similar to those presented for pre-let-7g in Figure 4 of the main text. (B) Gel showing the effects of omitting reverse transcriptase (RT) from PCR reactions used to detect pre-let-7g in α-RBM3 immunoprecipitate and input fractions. The pre-let-7g product is only amplified in each fraction if RT is included. (PDF) [file pone.0028446.s007.pdf]
