# Supplementary material for: Widespread Regulation of miRNA Biogenesis at the Dicer Step by the Cold-Inducible RNA-Binding Protein, RBM3
Source: PLoS One. 2011 Dec 1;6(12):e28446. doi: 10.1371/journal.pone.0028446 (PMC3228759; doi:10.1371/journal.pone.0028446)
Supplement: Table S1 — Agilent miRNA array data. Table listing 159 miRNAs showing significant expression changes, out of 216 detected above threshold using Agilent miRNA microarrays, after knockdown of RBM3 in the B104 neuronal cell line. (PDF) [file pone.0028446.s008.pdf]

Supplementary Table SI: Agilent miRNA Array Data

miRNAs significantly changed after knockdown of RBM3 in the B104 cell line (FC = Fold Change)						
miRNA ID	log FC	FC	abs(FC)	Ave Expr	P Value	Adj.P Value
mmu-miR-125b-5p	-2.614	0.163	6.121	10.808	5.32E-05	0.00192
mmu-miR-199b*	-2.489	0.178	5.614	7.539	9.14E-05	0.00277
mmu-miR-199b	-2.335	0.198	5.045	7.566	2.16E-04	0.00400
mmu-miR-125a-5p	-2.307	0.202	4.949	7.407	3.39E-05	0.00169
mmu-miR-93	-2.046	0.242	4.129	8.753	1.18E-04	0.00335
mmu-miR-374	-1.952	0.258	3.869	7.072	2.08E-04	0.00400
mmu-miR-152	-1.936	0.261	3.825	7.612	3.82E-04	0.00580
mmu-miR-338-3p	-1.897	0.268	3.725	8.621	4.10E-04	0.00606
mmu-miR-15b	-1.863	0.275	3.637	8.948	6.40E-04	0.00665
mmu-miR-16	-1.841	0.279	3.582	9.888	6.38E-04	0.00665
mmu-miR-10a	-1.827	0.282	3.547	7.443	4.49E-04	0.00632
mmu-miR-103	-1.821	0.283	3.534	8.478	4.71E-04	0.00647
mmu-miR-130a	-1.814	0.284	3.515	9.307	7.47E-04	0.00684
mmu-let-7i	-1.780	0.291	3.434	10.353	1.15E-03	0.00803
mmu-let-7c	-1.768	0.294	3.406	11.017	5.94E-04	0.00665
mmu-miR-455	-1.752	0.297	3.369	7.518	7.27E-05	0.00233
mmu-miR-9	-1.717	0.304	3.288	7.842	7.12E-04	0.00671
mmu-miR-29c	-1.714	0.305	3.280	8.918	8.45E-04	0.00684
mmu-miR-7a	-1.712	0.305	3.276	6.343	5.16E-04	0.00649
mmu-miR-29b	-1.702	0.307	3.253	10.590	1.23E-03	0.00826
mmu-miR-10b	-1.689	0.310	3.224	6.625	6.07E-04	0.00665
mmu-miR-99b	-1.685	0.311	3.216	7.210	2.22E-04	0.00400
mmu-let-7b	-1.671	0.314	3.184	10.794	1.03E-03	0.00732
mmu-miR-24	-1.670	0.314	3.183	11.130	5.14E-04	0.00649
mmu-miR-18a	-1.665	0.315	3.170	7.164	7.65E-04	0.00684
mmu-miR-25	-1.655	0.317	3.150	8.936	1.65E-03	0.01021
mmu-let-7a	-1.644	0.320	3.125	10.940	1.89E-03	0.01125
mmu-miR-30b	-1.642	0.321	3.120	7.713	6.75E-04	0.00671
mmu-miR-107	-1.640	0.321	3.117	8.069	9.34E-04	0.00719
mmu-miR-106b	-1.630	0.323	3.095	8.907	8.06E-04	0.00684
mmu-miR-455*	-1.628	0.324	3.090	6.221	5.69E-04	0.00665
mmu-miR-106a	-1.619	0.325	3.072	7.603	3.43E-04	0.00550
mmu-miR-17	-1.605	0.329	3.042	7.718	3.73E-04	0.00580
mmu-miR-27b	-1.601	0.330	3.034	8.993	5.28E-04	0.00649
mmu-miR-26a	-1.601	0.330	3.034	10.082	7.18E-04	0.00671
mmu-let-7g	-1.578	0.335	2.986	9.095	2.31E-03	0.01160
mmu-miR-19b	-1.564	0.338	2.956	9.755	1.61E-03	0.01008
mmu-miR-30a	-1.562	0.339	2.953	7.735	1.48E-03	0.00949
mmu-let-7d	-1.560	0.339	2.949	9.765	1.97E-03	0.01133
mmu-miR-193	-1.557	0.340	2.943	7.553	6.92E-04	0.00671
mmu-miR-26b	-1.544	0.343	2.916	7.861	8.54E-04	0.00684
mmu-let-7f	-1.531	0.346	2.890	10.784	2.25E-03	0.01147
mmu-miR-20a	-1.518	0.349	2.863	9.501	2.07E-03	0.01133
mmu-miR-20b	-1.505	0.352	2.837	8.979	2.50E-03	0.01211
mmu-miR-324-5p	-1.494	0.355	2.817	6.772	1.23E-03	0.00826
mmu-miR-19a	-1.490	0.356	2.810	7.770	2.17E-03	0.01133
mmu-miR-363	-1.484	0.357	2.798	7.779	2.56E-03	0.01231
mmu-miR-652	-1.476	0.360	2.781	6.118	5.22E-04	0.00649

mmu-miR-92a	-1.455	0.365	2.741	8.159	2.59E-04	0.00439
mmu-let-7e	-1.436	0.370	2.706	8.275	2.16E-03	0.01133
mmu-miR-210	-1.435	0.370	2.705	8.419	4.79E-03	0.01853
mmu-miR-361	-1.434	0.370	2.702	6.150	2.15E-03	0.01133
mmu-miR-298	-1.426	0.372	2.686	6.278	4.91E-05	0.00192
mmu-miR-301a	-1.421	0.373	2.677	7.716	3.85E-03	0.01609
mmu-miR-196a	-1.415	0.375	2.666	6.743	1.50E-03	0.00949
mmu-miR-532-3p	-1.409	0.376	2.656	5.630	4.20E-04	0.00606
mmu-miR-362-5p	-1.389	0.382	2.620	6.302	7.82E-04	0.00684
mmu-miR-193b	-1.388	0.382	2.617	5.612	9.14E-04	0.00713
mmu-miR-100	-1.387	0.382	2.615	6.467	1.94E-03	0.01133
mmu-miR-15a	-1.383	0.383	2.609	8.478	1.44E-03	0.00947
mmu-miR-30c	-1.363	0.389	2.573	7.233	7.95E-04	0.00684
mmu-miR-33	-1.363	0.389	2.571	5.996	3.52E-05	0.00169
mmu-miR-29a	-1.351	0.392	2.551	11.093	5.24E-03	0.01976
mmu-miR-27a	-1.341	0.395	2.534	10.609	4.43E-03	0.01741
mmu-miR-98	-1.340	0.395	2.532	7.433	3.36E-03	0.01456
mmu-miR-350	-1.330	0.398	2.515	6.342	2.10E-03	0.01133
mmu-miR-9*	-1.329	0.398	2.513	6.734	2.19E-03	0.01133
mmu-miR-34a	-1.315	0.402	2.487	8.847	4.38E-03	0.01741
mmu-miR-101a	-1.300	0.406	2.463	6.049	6.98E-04	0.00671
mmu-miR-24-2*	-1.287	0.410	2.440	6.389	6.46E-04	0.00665
mmu-miR-149	-1.285	0.410	2.437	7.638	6.00E-04	0.00665
mmu-miR-362-3p	-1.283	0.411	2.434	7.273	3.15E-03	0.01383
mmu-miR-34c	-1.283	0.411	2.434	7.907	4.44E-03	0.01741
mmu-miR-23b	-1.265	0.416	2.403	9.520	1.34E-03	0.00886
mmu-miR-221	-1.257	0.418	2.390	8.425	3.78E-03	0.01593
mmu-miR-365	-1.250	0.420	2.378	7.867	3.59E-03	0.01525
mmu-miR-345-5p	-1.248	0.421	2.375	6.235	6.20E-04	0.00665
mmu-miR-34b-5p	-1.245	0.422	2.371	8.139	5.17E-03	0.01962
mmu-miR-425	-1.236	0.424	2.356	6.211	2.20E-03	0.01133
mmu-miR-30e	-1.227	0.427	2.340	7.129	2.42E-03	0.01189
mmu-miR-340-5p	-1.220	0.429	2.329	6.011	1.04E-03	0.00732
mmu-miR-21	-1.199	0.436	2.295	12.929	1.24E-02	0.03782
mmu-miR-31	-1.190	0.438	2.282	7.702	1.05E-02	0.03298
mmu-miR-151-5p	-1.185	0.440	2.273	6.595	6.88E-03	0.02405
mmu-miR-140	-1.182	0.441	2.269	6.259	2.18E-03	0.01133
mmu-miR-101b	-1.175	0.443	2.258	6.220	2.64E-03	0.01259
mmu-miR-497	-1.160	0.447	2.235	5.859	3.60E-03	0.01525
mmu-miR-204	-1.143	0.453	2.209	7.243	3.97E-03	0.01649
mmu-miR-128	-1.139	0.454	2.202	6.748	7.20E-03	0.02459
mmu-miR-30d	-1.137	0.455	2.199	7.098	5.48E-04	0.00659
mmu-miR-674*	-1.120	0.460	2.174	5.578	3.15E-03	0.01383
mmu-miR-342-3p	-1.102	0.466	2.146	6.173	2.11E-03	0.01133
mmu-miR-148a	-1.100	0.466	2.144	5.491	2.96E-03	0.01355
mmu-miR-331-3p	-1.095	0.468	2.136	5.990	1.66E-03	0.01021
mmu-miR-132	-1.057	0.481	2.081	4.870	1.94E-03	0.01133
mmu-miR-22	-1.040	0.486	2.056	9.856	7.50E-03	0.02501
mmu-miR-23a	-1.039	0.487	2.055	11.413	9.59E-03	0.03059
mmu-miR-148b	-1.013	0.496	2.018	6.230	2.31E-03	0.01160
mmu-miR-195	-1.011	0.496	2.015	5.821	4.32E-03	0.01741

mmu-miR-378	-0.990	0.504	1.985	7.654	2.06E-03	0.01133
mmu-miR-146a	-0.974	0.509	1.964	7.137	5.36E-03	0.02007
mmu-miR-181d	-0.967	0.512	1.955	6.250	2.78E-03	0.01302
mmu-miR-744	-0.962	0.513	1.948	5.111	2.35E-04	0.00411
mmu-miR-99a	-0.954	0.516	1.937	5.167	3.16E-03	0.01383
mmu-miR-423-5p	-0.947	0.519	1.927	5.864	4.98E-03	0.01904
mmu-miR-185	-0.919	0.529	1.891	5.294	2.04E-03	0.01133
mmu-miR-872	-0.907	0.533	1.875	5.748	5.94E-03	0.02169
mmu-miR-335-5p	-0.902	0.535	1.869	5.857	1.47E-02	0.04357
mmu-miR-31*	-0.891	0.539	1.854	5.912	9.73E-03	0.03068
mmu-miR-325	-0.869	0.548	1.826	4.543	8.11E-04	0.00684
mmu-miR-324-3p	-0.868	0.548	1.825	4.959	9.78E-04	0.00732
mmu-miR-219	-0.867	0.548	1.823	5.730	7.60E-03	0.02516
mmu-miR-196b	-0.857	0.552	1.811	5.837	2.92E-03	0.01346
mmu-miR-326	-0.838	0.560	1.787	4.408	8.89E-04	0.00703
mmu-miR-106b*	-0.828	0.563	1.775	4.723	1.86E-04	0.00400
mmu-miR-140*	-0.824	0.565	1.770	7.098	1.54E-02	0.04451
mmu-miR-296-5p	-0.820	0.566	1.766	7.419	2.80E-03	0.01302
mmu-miR-181c	-0.812	0.570	1.755	5.608	7.40E-03	0.02482
mmu-miR-34c*	-0.806	0.572	1.749	4.659	1.41E-02	0.04207
mmu-miR-532-5p	-0.756	0.592	1.689	5.333	9.61E-04	0.00730
mmu-miR-7a*	-0.689	0.620	1.612	4.751	5.79E-03	0.02128
mmu-miR-218	-0.684	0.623	1.606	5.313	7.63E-03	0.02516
mmu-miR-34b-3p	-0.680	0.624	1.602	5.112	4.28E-03	0.01741
mmu-miR-18b	-0.662	0.632	1.583	4.920	4.39E-03	0.01741
mmu-miR-22*	-0.619	0.651	1.535	5.470	7.31E-03	0.02467
mmu-miR-24-1*	-0.617	0.652	1.534	4.698	2.71E-03	0.01280
mmu-miR-339-5p	-0.601	0.659	1.517	3.606	1.53E-02	0.04451
mmu-miR-186	-0.598	0.661	1.513	5.738	1.07E-02	0.03332
mmu-miR-320	-0.589	0.665	1.504	6.269	6.71E-03	0.02359
mmu-miR-197	0.587	1.502	1.502	4.217	6.00E-03	0.02177
mmu-miR-487b	0.600	1.516	1.516	6.708	7.02E-03	0.02424
mmu-miR-705	0.601	1.516	1.516	5.847	9.98E-04	0.00732
mmu-miR-762	0.618	1.534	1.534	4.122	3.16E-03	0.01383
mmu-miR-467c	0.662	1.582	1.582	4.920	2.21E-04	0.00400
mmu-miR-714	0.665	1.585	1.585	3.786	3.08E-03	0.01383
mmu-miR-328	0.672	1.593	1.593	3.967	2.43E-03	0.01189
mmu-miR-709	0.674	1.596	1.596	9.721	1.24E-02	0.03782
mmu-miR-290-5p	0.686	1.609	1.609	5.548	1.94E-04	0.00400
mmu-miR-669b	0.703	1.628	1.628	4.778	8.47E-04	0.00684
mmu-miR-297a	0.742	1.673	1.673	5.006	3.02E-04	0.00498
mmu-miR-134	0.763	1.697	1.697	5.152	1.32E-04	0.00346
mmu-miR-711	0.778	1.715	1.715	4.863	1.22E-04	0.00335
mmu-miR-877*	0.798	1.738	1.738	5.251	4.74E-03	0.01847
mmu-miR-680	0.877	1.837	1.837	5.495	1.49E-05	0.00169
mmu-miR-294	0.904	1.871	1.871	5.131	1.94E-04	0.00400
mmu-miR-877	0.908	1.876	1.876	5.491	2.73E-05	0.00169
mmu-miR-710	0.930	1.905	1.905	5.027	5.01E-05	0.00192
mmu-miR-207	1.054	2.076	2.076	4.413	1.53E-04	0.00376
mmu-miR-466h	1.086	2.123	2.123	5.069	1.57E-04	0.00376
mmu-miR-483	1.096	2.138	2.138	7.026	4.49E-05	0.00192

mmu-miR-135a*	1.104	2.150	2.150	6.113	3.37E-05	0.00169
mmu-miR-125a-3p	1.122	2.177	2.177	6.670	8.79E-06	0.00169
mmu-miR-671-5p	1.187	2.277	2.277	6.772	1.58E-05	0.00169
mmu-miR-672	1.231	2.347	2.347	5.302	1.63E-04	0.00376
mmu-miR-669a	1.302	2.465	2.465	5.422	3.05E-05	0.00169
mmu-miR-1224	1.514	2.855	2.855	11.719	2.36E-05	0.00169
mmu-miR-466c-5p	1.518	2.865	2.865	5.550	6.26E-06	0.00169
mmu-miR-468	1.618	3.068	3.068	5.474	1.89E-05	0.00169
mmu-miR-669c	1.712	3.277	3.277	6.035	1.15E-05	0.00169
mmu-miR-574-5p	2.272	4.830	4.830	7.052	5.73E-05	0.00195
