# Supplementary material for: Widespread Regulation of miRNA Biogenesis at the Dicer Step by the Cold-Inducible RNA-Binding Protein, RBM3
Source: PLoS One. 2011 Dec 1;6(12):e28446. doi: 10.1371/journal.pone.0028446 (PMC3228759; doi:10.1371/journal.pone.0028446)
Supplement: Table S2 — Oligonucleotides used. Table listing oligonucleotides used in Northern blot, in situ hybridization, RT-PCR, gel shift, processing, and pull-down assays, and siRNAs used to knockdown RBM3. (PDF) [file pone.0028446.s009.pdf]

Supplementary Table II: Oligonucleotides Used

Northern probes	
5S	5'-ATCTCGTCTGATCTCGGAAGCTAAGCAGGGTC GGGCCTGCCTGTCTC-3'
Let-7a	5'-TGAGGTAGTAGTGTTGTATAGTTCCTGTCTC-3'
Let-7g	5'-TGAGGTAGTAGTTTGTACAGTTCCTGTCTC-3'
Let-7i	5'-TGAGGTAGTAGTTTGTGCTGTTCCTGTCTC-3'
Mir-9	5'-TCTTTGGTTATCTAGCTGTATGACTGTCTC-3'
Mir-16	5'-TAGCAGCACGTAAATATTGGCGCCTGTCTC-3'
Mir-18a	5'-TAAGGTGCATCTAGTGCAGATAGCCTGTCTC-3'
Mir-24	5'-TGGCTCAGTTCAGCAGGAACAGCCTGTCTC-3'
Mir-30a	5'-TGTAAACATCCTCGACTGGAAGCTGTCTC-3'
Mir-30b	5'-TGTAAACATCCTACACTCAGCTCTGTCTC-3'
Mir-93	5'-CAAAGTGCTGTTCGTGCAGGTAGCCTGTCTC-3'
Mir-132	5'-TAACAGTCTACAGCCATGGTCGCCTGTCTC-3'
Mir-125a5p	5'-TCCCTGAGACCCTTTAACCTGTGACCTGTCTC-3'
Mir-1224	5'-GTGAGGACTGGGGAGGTGGAGCCTGTCTC-3'
pre-miR-125a	5'-GGC TCC CAA GAA CCT CAC CTG TGA CCC TGG ACG TCC TCA CAG GTT AAA GGG TCTCAG GGA-3'

ISH LNA probes	
Mmu-pre-let-7g	5'-AACTGTACAAACTACTACCTCA-3'
Mmu-let-7g	5'-CTGTACCGGGTGGTATCATAGA-3'

RT-PCR primers	
Pre-let-7g forward	5'-GTAGTAGTTTGTACAGTTTGAGGGT-3'
Pre-let-7g reverse	5'-GGCAGTGGCCTGTACAGT-3'
Pre-mir-16 forward	5'-GCAGCACGTAAATATTGGCGT-3'
Pre-mir-16 reverse	5'-AAGCAGCACAATAATATTGGTGT-3'
β-tubulin forward	5'-TGTCTACTACAATGAGGCCTCC-3'

β-tubulin reverse	5'-CTCACTATAGGGCTGACCGAAGATAAAGT TGTCAG-3'

Gel Shift / Processing / Biotin probes	
Pre-let-7g	5'-CCTGGCAAGGCAGTGGCCTGTACAGTTATCTCCTG TACCGGGTGGTATCATAGACCCTCAAACTGTACAAAC TACTACCTCAGCCTGGCCTGTCTC-3'
Pre-mir-16	5'-AACCTTACTTCAGCAGCACAGTCAATACTGGAGGT AATTTCAGAATCTTAACGCCAATATTTACGTGCTGCT AAGGCACCGCTCTGTCTC-3'

RBM3 siRNA	
siRBM3 sense	5'-CCUUCACAAACCCAGAGCATT-3'
siRBM3 antisense	5'-UGCUCUGGGUUUGUGAAGGTG-3'
